# Exploring the relative efficacy of motion artefact correction techniques for EEG data acquired during simultaneous fMRI

**DOI:** 10.1002/hbm.24396

**Published:** 2018-10-19

**Authors:** Alexander J. Daniel, James A. Smith, Glyn S. Spencer, João Jorge, Richard Bowtell, Karen J. Mullinger

**Affiliations:** ^1^ Sir Peter Mansfield Imaging Centre, School of Physics and Astronomy University of Nottingham, University Park Nottingham United Kingdom; ^2^ Department of Physics Loughborough University Leicestershire United Kingdom; ^3^ Laboratory for Functional and Metabolic Imaging École Polytechnique Fédérale de Lausanne Lausanne Switzerland; ^4^ Birmingham University Imaging Centre, School of Psychology University of Birmingham Birmingham United Kingdom

**Keywords:** artefact correction, head motion artefact, motion artefact detection, quantitative comparison, simultaneous EEG‐fMRI

## Abstract

Simultaneous EEG‐fMRI allows multiparametric characterisation of brain function, in principle enabling a more complete understanding of brain responses; unfortunately the hostile MRI environment severely reduces EEG data quality. Simply eliminating data segments containing gross motion artefacts [MAs] (generated by movement of the EEG system and head in the MRI scanner's static magnetic field) was previously believed sufficient. However recently the importance of removal of *all* MAs has been highlighted and new methods developed. A systematic comparison of the ability to remove MAs and retain underlying neuronal activity using different methods of MA detection and post‐processing algorithms is needed to guide the neuroscience community. Using a head phantom, we recorded MAs while simultaneously monitoring the motion using three different approaches: Reference Layer Artefact Subtraction (RLAS), Moiré Phase Tracker (MPT) markers and Wire Loop Motion Sensors (WLMS). These EEG recordings were combined with EEG responses to simple visual tasks acquired on a subject outside the MRI environment. MAs were then corrected using the motion information collected with each of the methods combined with different analysis pipelines. All tested methods retained the neuronal signal. However, often the MA was not removed sufficiently to allow accurate detection of the underlying neuronal signal. We show that the MA is best corrected using the RLAS combined with post‐processing using a multichannel, recursive least squares (M‐RLS) algorithm. This method needs to be developed further to enable practical utility; thus, WLMS combined with M‐RLS currently provides the best compromise between EEG data quality and practicalities of motion detection.

## 1 INTRODUCTION

Simultaneous EEG‐fMRI is a multimodal technique that has been widely exploited in the investigation of brain function. The combination of these modalities in simultaneous EEG‐fMRI recordings has shown great utility in the investigation of unpredictable brain responses. Simultaneous EEG‐fMRI has primarily been used to relate electrophysiological and haemodynamic measures of brain activity made during spontaneous changes in brain state (i) at rest (e.g., Goldman, Stern, Engel, & Cohen, 2002; Laufs et al., 2003), (ii) during sleep (e.g., Horovitz et al., 2008; Wilson et al., 2015) or (iii) due to pathology, such as epilepsy (e.g., Salek‐Haddadi, Merschhemke, Lemieux, & Fish, 2002; Pittau, Dubeau, & Gotman, 2012; Masterton, Jackson, & Abbott, 2013); or in single‐trial responses to sensory, motor or cognitive tasks (e.g., Debener et al., 2005; Eichele et al., 2005; Ritter, Moosmann, & Villringer, 2009; Mayhew, Dirckx, Niazy, Iannetti, & Wise, 2010; Mayhew, Ostwald, Porcaro & Bagshaw, 2013; Mullinger, Mayhew, Bagshaw, Bowtell, & Francis, 2014; Sadaghiani et al., 2010). This has provided new insight into the origin of neural oscillations (e.g., Goldman et al., 2002; Laufs et al., 2003; Scheeringa, Koopmans, van Mourik, Jensen, & Norris, 2016), the origin of haemodynamic responses and the role of neurovascular coupling (e.g., Mayhew et al., 2013; Mullinger, Mayhew, Bagshaw, Bowtell, & Francis, 2013; Mullinger, Cherukara, Buxton, Francis, & Mayhew, 2017). In addition it has been shown that simultaneous EEG‐fMRI can provide greater specificity regarding the temporal sequence (Eichele et al., 2005; Mayhew, Li, & Kourtzi, 2012) of activity in responsive brain areas, compared with that provided by standard analysis of single‐modality neuroimaging data.

The benefits of simultaneous EEG‐fMRI are therefore clear, but technical challenges still hamper its use. These challenges primarily relate to the EEG data quality, which is severely affected by the hostile electromagnetic environment inside an MRI scanner. There are three main artefacts which are induced in the EEG data: (1) the gradient artefact (GA), caused by the switching of magnetic field gradients that are required in MRI (Yan, Mullinger, Brookes, & Bowtell, 2009); (2) the pulse artefact (PA), related to the cardiac cycle and related pulsatile blood flow, thought to be induced by head motion and blood movement in the large static magnetic field of the MRI scanner (Yan, Mullinger, Geirsdottir, & Bowtell, 2010); (3) motion artefact (MA) caused by voluntary or involuntary head motion which results in the movement of the conductive paths of the EEG system and head in the static magnetic field (Jansen et al., 2012). In addition to these effects other sources such as the helium pumps, ventilation, and lights can add additional noise into the EEG data acquired in the MRI environment (Mullinger, Brookes, Stevenson, Morgan, & Bowtell, 2008), but these effects can usually be overcome by switching off these noise sources.

While considerable effort has been applied to removing the GA and PA via reduction of the strength of the artefacts produced during acquisition (e.g., Bonmassar et al., 2002; Chowdhury, Mullinger, & Bowtell, 2015; Chowdhury, Mullinger, Glover, & Bowtell, 2014; Jorge, Grouiller, Gruetter, van der Zwaag, & Figueiredo, 2015; LeVan et al., 2013; Luo, Huang, & Glover, 2014; Maziero et al., 2016; Mullinger, Chowdhury, & Bowtell, 2014; Mullinger, Yan, & Bowtell, 2011; Solana et al., 2014; Steyrl, Krausz, Koschutnig, Edlinger, & Muller‐Putz, 2017) and application of post‐processing methods (e.g., Abreu et al., 2016; Acharjee, Phlypo, Wu, Calhoun, & Adali, 2015; Allen, Josephs, & Turner, 2000; Allen, Polizzi, Krakow, Fish, & Lemieux, 1998; Bonmassar et al., 2002; Brookes, Mullinger, Stevenson, Morris, & Bowtell, 2008; De Munck, van Houdt, Goncalves, van Wegen, & Ossenblok, 2013; Iannotti, Pittau, Michel, Vulliemoz, & Grouiller, 2015; Krishnaswamy et al., 2016; Luo, Huang, & Glover, 2014; Niazy, Beckmann, Iannetti, Brady, & Smith, 2005; Xia, Ruan, & Cohen, 2014), until recently, little attention had been given to removing the MA. This is because it was thought that the identification of gross MAs, via data inspection, followed by removal of confounded data segments, produced EEG data of high enough quality to use in EEG‐fMRI data analysis pipelines (Allen et al., 1998). However, recent studies have highlighted the problems of this approach, showing that small MAs remain which can dominate the EEG signals of interest, even when stringent post‐processing pipelines to remove MAs are employed (Fellner et al., 2016; Jansen et al., 2012). The greatest problem is that the MA is entirely unpredictable both temporally and in spatial topology (Fellner et al., 2016; Jansen et al., 2012; Jorge et al., 2015; Masterton, Abbott, Fleming, & Jackson, 2007; Maziero et al., 2016). MAs can produce physiologically plausible patterns of EEG activity (Fellner et al., 2016) that may be temporally correlated with BOLD responses (Fellner et al., 2016; Jansen et al., 2012), making improved MA correction strategies vital for the advancement of EEG‐fMRI application in neuroscience.

The problem of MA contamination in EEG data is now well accepted and has resulted in the development of a number of different methods for removing the MAs from EEG data through the monitoring of head movement. An early approach (Bonmassar et al., 2002; Hill, Chiappa, Huanghellinger, & Jenkins, 1995) involved detecting and correcting MAs using a piezoelectric sensor that was attached to the head. This approach has not been widely adopted, perhaps due to the need for a piezoelectric device which does not create MRI artefacts, and which is not detrimentally affected by GAs. In addition the piezoelectric sensor is sensitive to all head movements including rigid body translations which do not necessarily generate EEG MAs.

Masterton et al. proposed an alternative method of monitoring head motion by measuring the voltages induced in a four carbon wire loops affixed to the EEG cap (Masterton et al., 2007). They showed that this method worked well for smaller head movements, but failed to remove the MAs in a subject making larger head movements of up to 10 mm in extent. They also showed, through simulation, that they could satisfactorily recover a 10 Hz sinusoidal signal (produced using a signal generator) from data confounded by MAs due to real head motion, using their wire‐loop MA correction method. Van der Meer et al. (2016) recently employed a similar carbon wire loop set‐up to show that artefacts related to the cardiac cycle and helium pumps could be better corrected using the wire loop method than was possible using three conventional post‐processing approaches. However, this study did not consider the efficacy for correcting MAs due to head motion. Jorge et al. (2015) adapted this method to use the leads and electrodes on a standard EEG cap to form wire loops, making implementation easier with a standard EEG system. They employed the same multi‐channel recursive least‐squares (M‐RLS) algorithm used by Masterton et al. (2007) to fit the data from the wire loops to the EEG channel data and correct the individual channels. This work, however, involved exclusion of segments of data recorded during gross movements, only assessing the efficacy of the method for removing the PA and smaller ongoing MAs.

In contrast, the reference layer artefact subtraction (RLAS) approach, which was introduced by Chowdhury et al. (2014), uses an entirely separate set of electrodes that are connected to a scalp‐shaped conducting layer to capture all artefacts including the MA. The signals measured from the electrodes on the reference layer are subtracted from the signals measured at the scalp electrodes to eliminate the artefacts (Chowdhury et al., 2014). This method has been extended by Steyrl et al. (2017), who produced a double‐layer cap in which the electrodes used to monitor motion are connected via a series of conductive tubes, rather than a continuous layer. Using this system, they showed that least‐mean squares adaptive filtering of the reference layer signals to the scalp layer produced superior performance to the simple subtraction used in the original RLAS implementation (Steyrl et al., 2017).

Moiré Phase Tracker (MPT) markers (Maclaren et al., 2012) have also been used to capture head motion for the purpose of EEG MA correction (LeVan et al., 2013; Maziero et al., 2016). A camera in the bore of the magnet tracks the motion of the marker with six degrees of freedom and a sampling rate of approximately 80 Hz, sufficient to capture head motion. The first implementation of this approach focused on the removal of the PA only (LeVan et al., 2013). However, subsequently, Maziero et al. investigated the efficacy of MPT for removing MAs (Maziero et al., 2016). The original motion parameters, along with their derivatives (velocities) and derivatives squared were fed into a general linear model to correct the MAs in the EEG data. This approach to MA correction has been tested in experiments in which head movements produced up to 10 mm of translation, 6° of rotation and 50 mm/s marker velocity. The results show that a large proportion of the MA can be removed with this technique (Maziero et al., 2016).

While all of these methods have shown success in removing the MA, it is currently unclear which is most effective. Hermans et al. (2016) performed a comparison of the performance of the double‐layer reference device (Guger Technologies OG Graz, Austria) and the carbon wire loops approach (Masterton et al., 2007). They found that the two methods showed comparable performance for removal of PA and MA. However, a direct quantitative comparison of the two methods was difficult as data were recorded in separate sessions using different EEG caps with different electrode designs. Comparison of the correction of MAs is particularly challenging with this set‐up, since producing identical head motion in two sessions is impossible, even for an experienced person. This is relevant because the induced MA is affected by the rate, direction, and amplitude of movement as well as the head orientation in the MRI scanner. Furthermore, the methods described above employ different algorithms for fitting the motion metrics to the EEG data. While it has been shown that underlying neuronal signals are present after MA correction using all methods, it is unclear whether over‐fitting of the data is occurring, especially in the cases where adaptive filtering is employed (Jorge et al., 2015; Masterton et al., 2007; Steyrl et al., 2017). Such over‐fitting may attenuate the neuronal signals of interest. However, to our knowledge, an evaluation of MA correction techniques using true neuronal signals as the gold standard to be recovered, has not been possible in previous studies as the actual form of the neuronal signals has been unknown.

Here, we aim to provide a quantitative assessment of the relative merits of the three main methods which have been proposed for MA correction of EEG data namely, use of: wire loop motion sensors (WLMS) (Jorge et al., 2015), the reference layer approach (RLAS) (Chowdhury et al., 2014) or MPT markers (Maziero et al., 2016). We aim to assess the efficacy of removal of the MA as well as the ability of each method to retain the underlying neuronal signal using exactly the same data in testing the three different approaches. We aim to use this assessment to provide guidance on the relative merits of the methods for MA correction in future studies.

## 2 METHODS

All EEG data were acquired using a 32 channel BrainAmp MR amplifier (Brain Products, Munich, Germany), using a 5 kHz sampling rate, and frequency range of 0.016–250 Hz, with a 30 dB roll‐off per octave at high frequency. MA recordings were made inside a 3 T Achieva MRI system (Philips Medical Systems, Best, The Netherlands). All data acquired on the human subject was done with approval of the local ethics committee and the study was conducted in accordance with the *Helsinki Declaration*. The subject gave written, informed consent.

Data for this study were acquired in two stages: (i) the EEG MAs and data for all accompanying motion‐monitoring methods were acquired on a head‐shaped phantom in the MRI scanner; (ii) EEG data were acquired on a human subject outside the MRI environment to provide a gold standard recording of underlying neuronal activity.

The standard EEG signal, *S*_R_, recorded during simultaneous EEG‐fMRI, can be represented by:

(1) $$S_{R}=S_{\text{neuronal}}+S_{\text{artefact}}+\text{noise}$$

where, *S*_neuronal_ is the neuronal signal of interest and *S*_artefact_ is the artefact signals caused by the MRI environment (normally this includes GA, PA and MA, but here *S*_artefact_ only comprises MAs). Noise represents interference other than the GA, PA and MA, and the intrinsic electrical noise. The EEG data from the phantom and subject were summed together, separately for each electrode. This provided an EEG dataset containing neuronal signals confounded by MA, where the underlying neuronal signals to be recovered after MA correction were known.

### 2.1 Data acquisition

#### 2.1.1 MA recordings

MAs were recorded on a head‐shaped phantom made of 4% kappa carrageenan in deionised water (95.5%) containing 0.5% NaCl, such that the phantom had similar conductive properties to the human head (Yan et al., 2009). A phantom was used to ensure that only the *S*_artefact_ signal was recorded in the MRI environment. Hardware for all three motion‐detection and correction methods to be tested (WLMS, RLAS and MPT) were applied to the phantom simultaneously.

**Figure 1:**
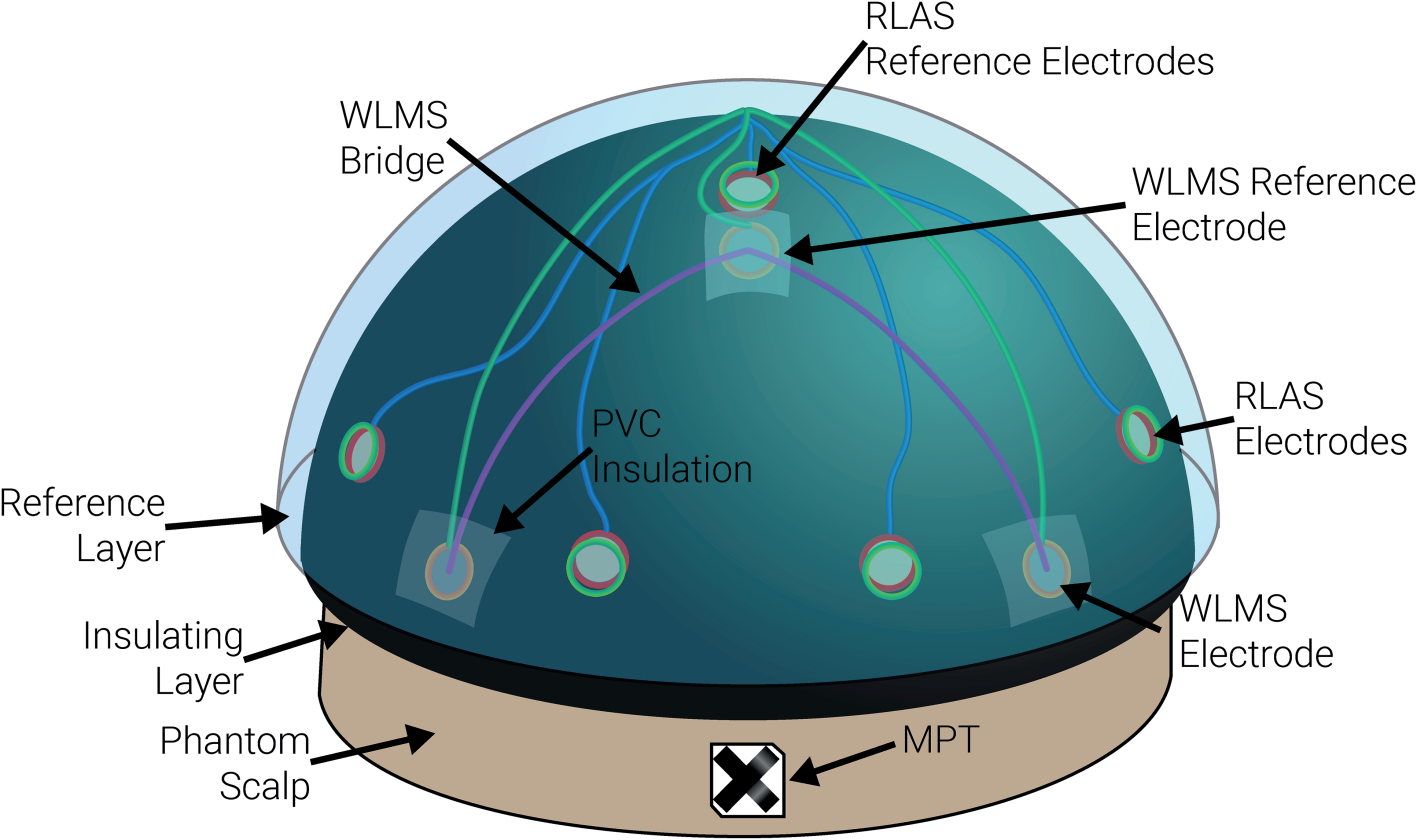
A schematic of the setup of the phantom used to record EEG MAs and simultaneously to collect motion data with the RLAS and WLMS systems and the MPT marker [Color figure can be viewed at http://wileyonlinelibrary.com]

A schematic of the EEG cap and associated motion tracking hardware can be seen in Figure 1. In detail, EEG data were recorded using a custom‐made RLAS EEG cap with nine scalp Ag/AgCl MRI‐compatible electrodes (EasyCap GmbH, Herrsching, Germany) at locations Fp1, Fp2, Fc5, Fc6, Cp5, Cp6, O1, Oz and O2. The reference electrode was positioned at Cz with the ground electrode at Pz. These electrode locations were chosen to provide an even coverage of the head locations where MAs are likely to be largest due to the area of the conductive loops formed by the reference electrode lead (at Cz), the head, and the recording electrodes. Leads (starquad cables [Van‐Damme Cable]) were bundled together where they left the EEG cap at the pole, producing a lead arrangement similar to that used in standard EEG caps. The scalp electrodes of the RLAS system were connected to the phantom using conductive gel and then sealed to provide electrical isolation from the reference layer. To implement the WLMS method: additional electrodes were attached to the surface of the insulating layer, at electrode locations F5, F6, T7 and T8, as used previously (Jorge et al., 2015). A separate reference electrode (to which the WLMS electrodes were re‐referenced during post‐processing [see below]), was positioned just in front of the RLAS reference electrode between Fz and Cz, and connected electrically to the scalp. Wire bridges were formed in an identical manner to that described in Jorge et al. (2015) to connect electrodes F5, F6, T7 and T8 to the corresponding reference electrode, thus forming four wire loops for MA detection. All of the WLMS electrodes were then insulated from the rest of the EEG set‐up using Polyvinyl Chloride (PVC) insulation tape. Conductive gel was placed into each of the RLAS reference layer electrodes and the conductive reference layer (made from hydrogel [Katecho, Inc., IA, USA]) applied. This reference layer covered a similar area to that of the insulating layer and extended under the chin region. It was tightly fitted to the phantom to prevent movement of this layer (or the WLMS) relative to the EEG electrodes. Finally, the MPT marker was attached to the phantom via toothpicks inserted into the forehead region of the phantom to simulate the rigid coupling of the MPT marker to the head that is usually achieved by mounting it on a bite‐bar (Maziero et al., 2016).

The phantom was placed in the MRI scanner inside a 32‐channel head RF coil (as is typically used for EEG‐fMRI recording) and all EEG electrodes (for RLAS and WLMS systems) were connected to the EEG amplifier via a cable bundle that ran through the length of the bore (~1.5 m) terminating in a breakout box. The amplifier sat outside the bore of the magnet on a table, and the cable bundle was attached to a cantilevered beam (Chowdhury et al., 2015) to isolate it from scanner vibrations. In separate recordings an investigator induced four types of motion, comprising small and large nodding and shaking movements, which are the gross movements most typically encountered in standard EEG‐fMRI experiments (nodding corresponding to a rotation of the phantom about a left–right axis and shaking corresponding to a rotation about a head‐foot axis). These movements were repeated continually in a cyclical fashion with an average frequency of 0.8 ± 0.2 Hz, for the time periods shown in Table 1 while data from the EEG scalp electrodes, the RLAS reference electrodes and the WLMS were recorded with BrainVision Recorder (v 1.2, Brain Products GmbH, Gilching, Germany). The MPT marker position was recorded using an MR compatible camera (Metria Innovation Inc., Milwaukee, USA) at sampling rate of approximately 80 Hz. No MRI acquisition occurred during these recordings, and the helium pumps were turned off (Mullinger, Castellone, & Bowtell, 2013) to minimise other sources of noise and so to provide as far as possible recording of pure MAs. To synchronise the data from the EEG and MPT recordings, a marker was output to both recording computers by the MRI scanner at the start and end of each recording period.

**Table 1:** The RMS amplitude of the translational displacements of the MPT and recording length of each of the movement types for each of the datasets

Dataset	Motion	RMS amplitude (mm)	Recording length (s)
1	Small nod	1.0	37
	Small shake	0.9	107
	Large nod	2.6	40
	Large shake	3.1	107
2	Small nod	1.9	868
	Small shake	1.3	889
	Large nod	7.3	871
	Large shake	5.9	876

Due to the complexity of the set‐up in which three different motion recording methods were recorded simultaneously, it was important to assess the consistency of results. Therefore, two datasets were recorded with this set‐up on two separate days, with the equipment being removed from, and then reapplied to, the phantom between sessions.

#### 2.1.2 Neuronal recordings

Additional data were recorded from a human subject outside the scanner to allow subsequent assessment of the effect of MA artefact correction on a “gold standard” neuronal signal (*S*_neuronal_, Equation 1). Data were collected using a standard 32‐channel MR‐compatible BrainCap (EasyCap GmbH, Herrsching, Germany). This EEG cap contained electrodes of identical composition (i.e., Ag/AgCl MRI‐compatible ring electrodes) to those in the RLAS cap. 31 of the electrodes followed the extended 10–20 system, with a reference electrode positioned between Fz and Cz, while an additional channel for electrooculography was connected to an electrode placed under the left eye.

To allow the ability to recover both oscillatory and evoked (event related potentials [ERPs]) neuronal responses to be tested, data were acquired on a single subject using two different paradigms. The subject was requested to sit in a comfortable chair and relax with a computer screen in front of them on which stimuli were presented.

The first paradigm was designed to modulate the oscillatory alpha rhythm (8–13 Hz). Data were acquired with the room lights off and a fixation cross on a grey background presented on the screen. The subject was cued to open and close their eyes (alternating) when they heard an auditory tone (1 kHz for 0.5 s) presented every 30–35 s, along with a visual instruction on the screen. A marker was placed in the EEG recording each time that the subject was cued to open/close their eyes. Five cycles of eyes open/closed (EOEC) data were acquired. This paradigm lasted approximately 6 min 20 s.

The second paradigm was designed to generate ERPs to allow assessment of the preservation of these signals at a single trial level, as well as in the average. Visual evoked potentials (VEPs) were generated by a single presentation of a 2 Hz radial checkerboard (i.e., a checkerboard presented for 0.5 s followed by contrast reversed version for 0.5 s). A rest period (grey screen with fixation cross) of 4–6 s (randomly jittered) was then provided before the next pair of checkerboards were presented. The subject was instructed to fixate on the cross presented at the centre of the screen at all times. A total of 120 blocks were presented resulting in 240 VEPs in total. A marker was placed in the EEG file from the presentation computer at every checkerboard stimulus presentation. This paradigm lasted approximately 13 min 40 s.

#### 2.1.3 Data combination

The neuronal data was processed on its own to provide a “gold standard” of expected neuronal activity for each paradigm. In addition, the neuronal EEG data from each paradigm was added to the corresponding EEG channels for each of the MA EEG datasets, for small/large amplitude head nod/shake. This resulted in a total of four datasets (corresponding to each motion type) for each of the two MA recording sessions and the “gold standard” dataset.

### 2.2 Data analysis

All processing was carried out in BrainVision Analyser 2.0 (Brain Products GmbH, Gilching, Germany) and MATLAB (The MathWorks Inc., Natick, USA). All data recorded with the EEG amplifier were down‐sampled to 500 Hz and filtered 0.02–80 Hz (8th order, zero‐order Butterworth filter) with a 50 Hz notch filter. MPT data were collected at 81.1 ± 13.4 Hz, this inconsistency in sample rate was due to limitations in hardware causing random small delays to frame sampling. However, a time stamp was provided with each frame sample, providing precise information on acquisition time and allowing the MPT data to be resampled to a constant frequency of 80 Hz before being up‐sampled to 500 Hz to match the sample rate of the EEG data. EEG data and MPT data were temporally aligned using the time stamp markers inserted in the datasets at the beginning and end of data acquisitions.

All data were visually inspected to ensure high data quality had been recorded on each channel. As a result, Fc5 had to be excluded from MA dataset 1, with no channels excluded for MA dataset 2. To ensure equivalence in comparing MA correction methods, only neuronal signals from electrodes [Fp1, Fp2, Fc6, Cp5, Cp6, O1, Oz and O2]/[Fp1, Fp2, Fc5, Fc6, Cp5, Cp6, O1, Oz and O2] were combined with MA datasets 1/2, respectively. To provide an estimate of the magnitude of movement for each of the MA datasets the root mean squared (RMS) displacement (estimated as $\sqrt{\left(\bar{x^{2}+y^{2}+z^{2}}\right)}$, where *x*, *y* and *z* represent the change in the MPT position parameters relative to the initial position) was calculated.

MA correction was then performed on each of the datasets that had been generated using the following methods.

#### 2.2.1 RLAS

For data collected using the RLAS system (Chowdhury et al., 2014), reference‐layer EEG channels were re‐referenced to the electrode paired with the scalp reference electrode that was used as the reference for all channels during the recording. Data for each channel were then baseline‐corrected by subtraction of the mean signal across all time.

The simplest artefact correction method then consisted of a subtraction of the signal from the reference layer electrode directly overlaying each of the scalp layer electrodes, as previously implemented (Chowdhury et al., 2014). Given the known discrepancy between the MAs induced on the scalp and reference layers (Spencer, Smith, Chowdhury, Bowtell, & Mullinger, 2018), a simple linear fit of each reference electrode signal to the corresponding scalp electrode signal was also performed. This fitting was performed with a least‐squares fit, which was non‐adaptive over the time‐course, minimising the chance of over‐fitting and consequent removal of neuronal signals of interest. An adaptive fit was also implemented on these data using the M‐RLS algorithm, originally applied to WLMS data by Masterton et al. (2007). The implementation of the M‐RLS algorithm and specific parameters used are described in the WLMS section, below.

#### 2.2.2 MPT

The MPT data were used to perform MA correction as described by Maziero et al. (2016). Briefly, MPT data were low‐pass filtered with an 11 Hz cut‐off frequency, and the derivatives (velocities) and derivatives squared (modelling non‐linearities related to velocity) were calculated. This gave a total of 18 MA measures, which were input into a general linear model design matrix and fitted to the EEG data from each scalp channel. After MA correction, the EEG data were filtered 0.5–40 Hz [matching the procedure used in Maziero et al. (2016)] before further qualitative and quantitative analysis. The M‐RLS fitting algorithm was also implemented using these MPT data (without the 11 Hz low‐pass filter) in conjunction with the scalp EEG data (see WLMS section for parameter details).

#### 2.2.3 WLMS

The WLMS data from channels F5, F6, T7 and T8 were first re‐referenced to the reference electrode created for the WLMS (Figure 1). The M‐RLS algorithm as described and implemented by Masterton et al. (2007) was employed using the WLMS data (filtered 0.02–80 Hz) to provide the estimates of the motion, as previously described by Jorge et al. (2015). The algorithm was initialised with the following parameters: adaptability factor (λ) = 1–10^−8^; initial filter weights (ω(0)) = 0 and initial inverse correlation matrix (***P***(0)) =1 × 10^−3^ I (where I is the identity matrix)**.** The filter length and down‐sampling factor were optimised by exploring a range of filter lengths between 0 and 35 samples [in increments of 1, where 35 had been used previously (Jorge et al., 2015; Masterton et al., 2007)] and down‐sampling factors between 1 and 15 [in increments of 1, where 2 had been used previously (Jorge et al., 2015; Masterton et al., 2007)]. This optimisation was done using 2 min 20 s of EOEC neuronal data combined with the small‐amplitude, head nod MA data. These data were then corrected with M‐RLS using each of the filter lengths and down‐sampling parameters for each channel of neuronal data. The correlation between the original neuronal signal and the artefact corrected signal, as well as the ratio of the root‐mean square amplitude of the original to corrected signal was assessed for each combination of filter length and down‐sampling factor to determine the best combination of parameters (see also “*Quantitative Assessment of data quality”* section below).

The WLMS data with the M‐RLS fitting algorithm performed very well in correcting MA from the EEG data. Therefore, to explore whether this performance was due to the WLMS data accurately capturing the MA, or the M‐RLS algorithm providing excellent fitting of motion data to the EEG data, the use of the M‐RLS algorithm with other measures of motion was also evaluated. The RLAS reference layer measures of motion artefact (9 motion signals) and subsequently the MPT (original, derivatives and derivatives squared, giving 18 motion signals) were input into the M‐RLS algorithm in place of the WLMS measures of motion artefact, using the same parameters in the algorithm as used for the WLMS M‐RLS correction. All motion measures when input into the M‐RLS had a 0.02–80 Hz filter applied rather than the specific filtering parameters for the different correction methods that are outlined in the sections (“RLAS” and “MPT”) above.

### 2.3 Assessing MA correction

#### 2.3.1 Oscillatory (EOEC) neuronal data

These data were segmented into eyes‐open and eyes‐closed epochs of 28 s duration (omitting the first and last second of the trial to avoid periods contaminated by eye movement and the auditory cue). Data epochs were Fourier transformed and averaged over eyes‐open and closed segments separately. The difference between these averaged power spectra (eyes‐closed to eyes‐open) was calculated to reveal a peak in the alpha band of the pure neuronal data recorded on the occipital electrodes (O1, Oz and O2). The same process was carried out for each movement type (small/large amplitude head nod/shake) and MA correction method to qualitatively assess the efficacy of the correction methods at revealing the underlying neuronal activity from the MAs.

#### 2.3.2 VEP neuronal data

These data were segmented into 450 ms epochs relative to the onset of each checkerboard and baseline correction over the entire time window applied. The mean VEP measured at each electrode was then found and the electrode eliciting the largest VEP (P100‐N150 peak‐to‐peak amplitude) was chosen for further interrogation. Plots of this mean VEP response for the original neuronal data and after correction of each type of MA (small/large amplitude head nod/shake) with each correction method were created, to allow visual comparison of the average responses. In addition, the data from all the trials were plotted in stack plots where colour indicated the voltage at each time point and trial to allow visual assessment of single trial responses for each correction method.

#### 2.3.3 Quantitative assessment of data quality

Three metrics were calculated to provide a quantitative assessment of the relative performance of each MA correction method for each movement type over all EEG channels. These metrics were derived for the oscillatory (EOEC) and evoked (VEP) data, separately. They were calculated over the entire time‐courses of the paradigms rather than only for the epochs that were used in the qualitative analyses.

The Pearson's correlation coefficient between each channel of the corrected data and its corresponding “gold standard” (i.e., the neuronal data before MA had been added) was calculated. This provided a measure of how well each method retained the shape of the original waveform. To assess whether the amplitude of the signal had also been retained, the ratio of the RMS calculated on the gold standard data to the RMS of MA‐corrected signals was also calculated. Finally, an estimate of the signal‐to‐noise ratio (SNR) was calculated using:

(2) $$\mathrm{SNR}=\left(\frac{\mathrm{RMS}\left(S_{\text{neuronal}}\right)}{\mathrm{RMS}\left(S_{\text{corrected}}-S_{\text{neuronal}}\right)}\right)$$

where, *S*_neuronal_ is the gold standard neuronal signal (as used in Equation 1) and *S*_corrected_ is the MA corrected signal (which in an ideal case would be identical to *S*_neuronal_ but otherwise any signal is assumed to be remaining MA, i.e., noise).

For each of these metrics the mean and standard deviation over channels was evaluated for each of the datasets.

## 3 RESULTS

### 3.1 Data quality and alignment

**Figure 2:**
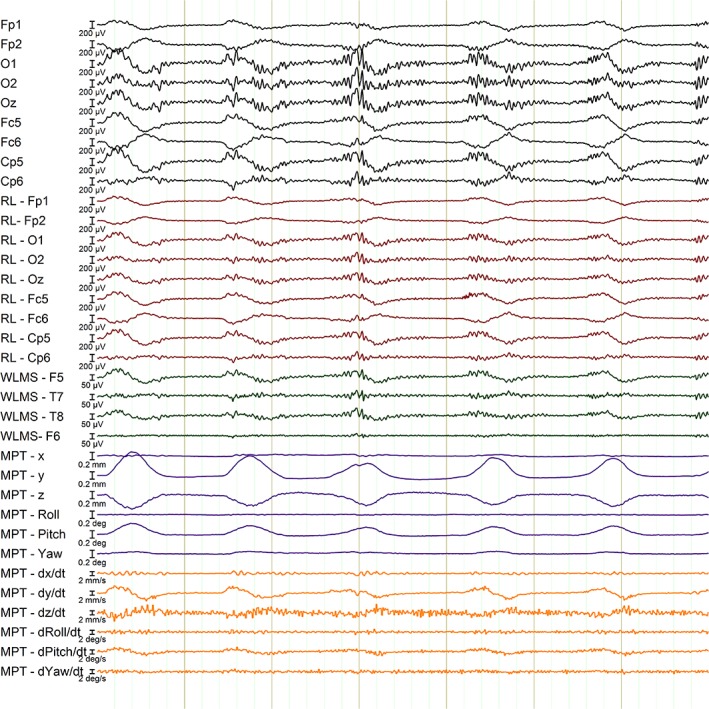
A 7 s segment of neuronal data (from the VEP paradigm) corrupted with MA from small amplitude head nods (black traces), with the corresponding channels detecting motion using different methods: RLAS – Red channels (from the reference layer); WLMS – Green channels (channels from the wire loops) and MPT – Purple channels (showing translations and rotations in approximately the MR scanner's reference frame where pitch denotes nodding action and roll denotes shaking action). The orange lines depict the variation with time of the temporal derivatives of the MPT measurements. RLAS and WLMS data are displayed after re‐referencing to their relevant reference. Note time between black vertical lines is 1 s [Color figure can be viewed at http://wileyonlinelibrary.com]

Good temporal alignment of the MPT and EEG data (and other motion measures) was achieved, as shown in Figure 2. The effect of the small‐amplitude head nods can be seen clearly as a MA in the EEG scalp channels (Figure 2, black traces) as well as in the motion detection methods (RLAS: red traces; WLMS: green traces; and MPT: purple traces). Note that the apparent temporal differences between the MPT traces and other data, occur because the MPT data represent measurements of displacement (translation and rotation), whereas the EEG MA relate to the rate of change of position (i.e., velocity), (orange traces).

The RMS of the motion for each of the datasets and movement types is shown in Table 1. As expected, the RMS values for the small movements were always substantially smaller than those for the large movements. However, the amplitude of the movements varied considerably between datasets, despite the experimenters visually monitoring the MPT marker displacement during data acquisition. This clearly illustrates the difficulty in maintaining a similar degree of movement across separate acquisitions, making it difficult to draw comparisons between the efficacy of different methods, when the movement data from different systems are not acquired simultaneously.

### 3.2 M‐RLS optimisation

**Figure 3:**
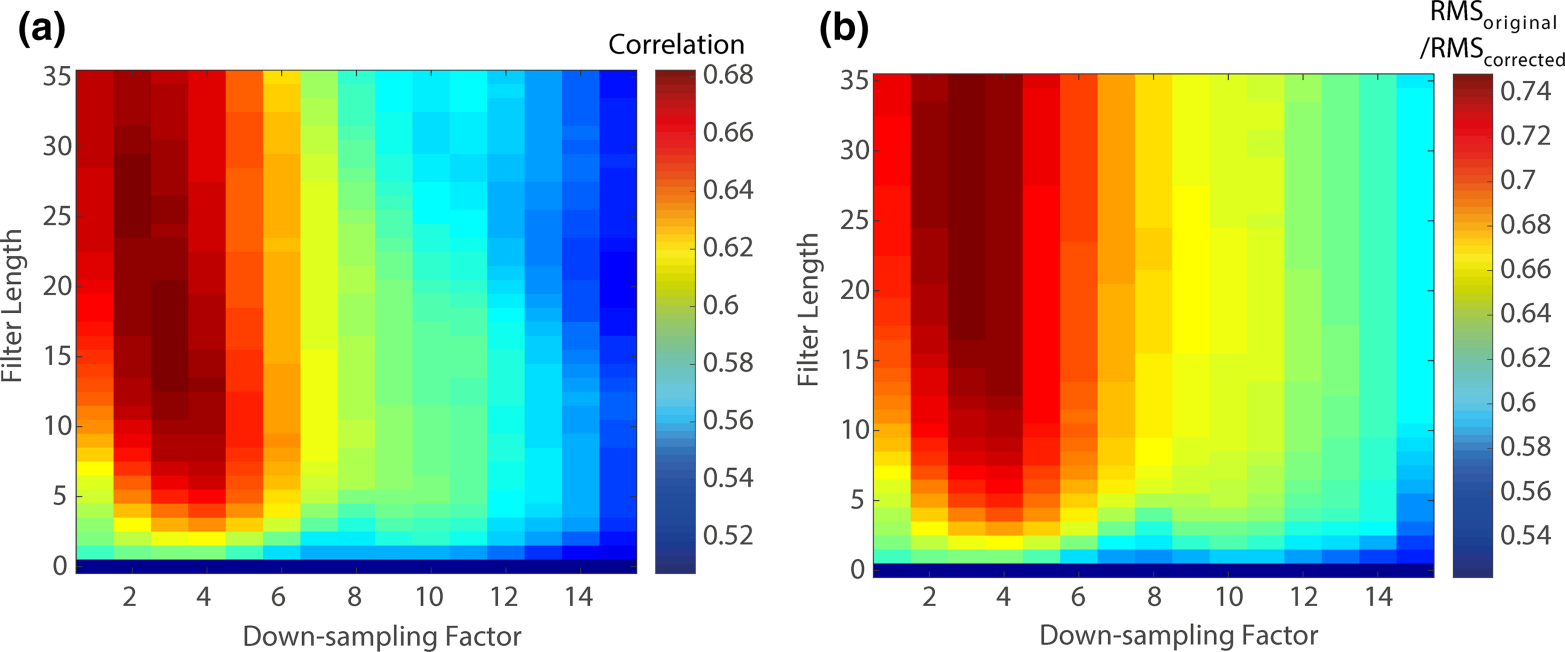
The effect of the filter length and down‐sampling factor on (a) the correlation between the gold standard (original) signal and the corrected signal and (b) the ratio of the RMS of the original and corrected signal. These plots show the average of each metric over all EEG channels using 2 min 20 s of neuronal data (from the VEP paradigm) with MA‐data from the small‐amplitude head nods added and subsequently corrected [Color figure can be viewed at http://wileyonlinelibrary.com]

The data that were used to ascertain the optimal filter length and down‐sampling factor parameters are shown in Figure 3. These plots clearly demonstrate the effects of both parameters on the correlation with the gold standard neuronal signal and the ratio of the RMS of the amplitude of the corrected signal to the gold standard. Variation of the down‐sampling factor has the most significant effect on these measures over the parameter space explored. High values of both these metrics indicate better performance within the scale range shown (note: if the RMS ratio exceeded 1 then this would indicate the MA correction was removing neuronal signals, which is obviously undesirable). There are practical benefits to limiting the filter length since the M‐RLS algorithm's execution time scales as the square of the filter length. We therefore chose a filter length of 15 and a down‐sampling factor of 3. These values gave the largest correlation value (Figure 3a) and a value of the RMS ratio which was 99.0% of the maximum value which occurred at a filter length of 32. The effect of the adaptability factor (λ) was also considered, as this parameter could also affect the performance of the M‐RLS algorithm: when the fitting weights change too quickly overfitting will result, while too slow changes will leave significant residual artefact in the MA‐corrected EEG data. However, within the range considered here (1–10^−4^ to 1–10^−12^), the filter length was found to have a far greater effect on the EEG data quality than the adaptability factor, as shown in Supporting Information Figure S1. Therefore, the previously used value of λ = 1–10^−8^ (Jorge et al., 2015; Masterton et al., 2007) was employed, along with a filter length = 15 and down‐sampling factor = 3, in all subsequent analyses using M‐RLS. An illustration of how the filter weights vary across reference layer leads and change over time for the small nod of the second dataset is shown in Supporting Information Figure S2.

### 3.3 Qualitative assessment of the oscillatory (EOEC) data

**Figure 4:**
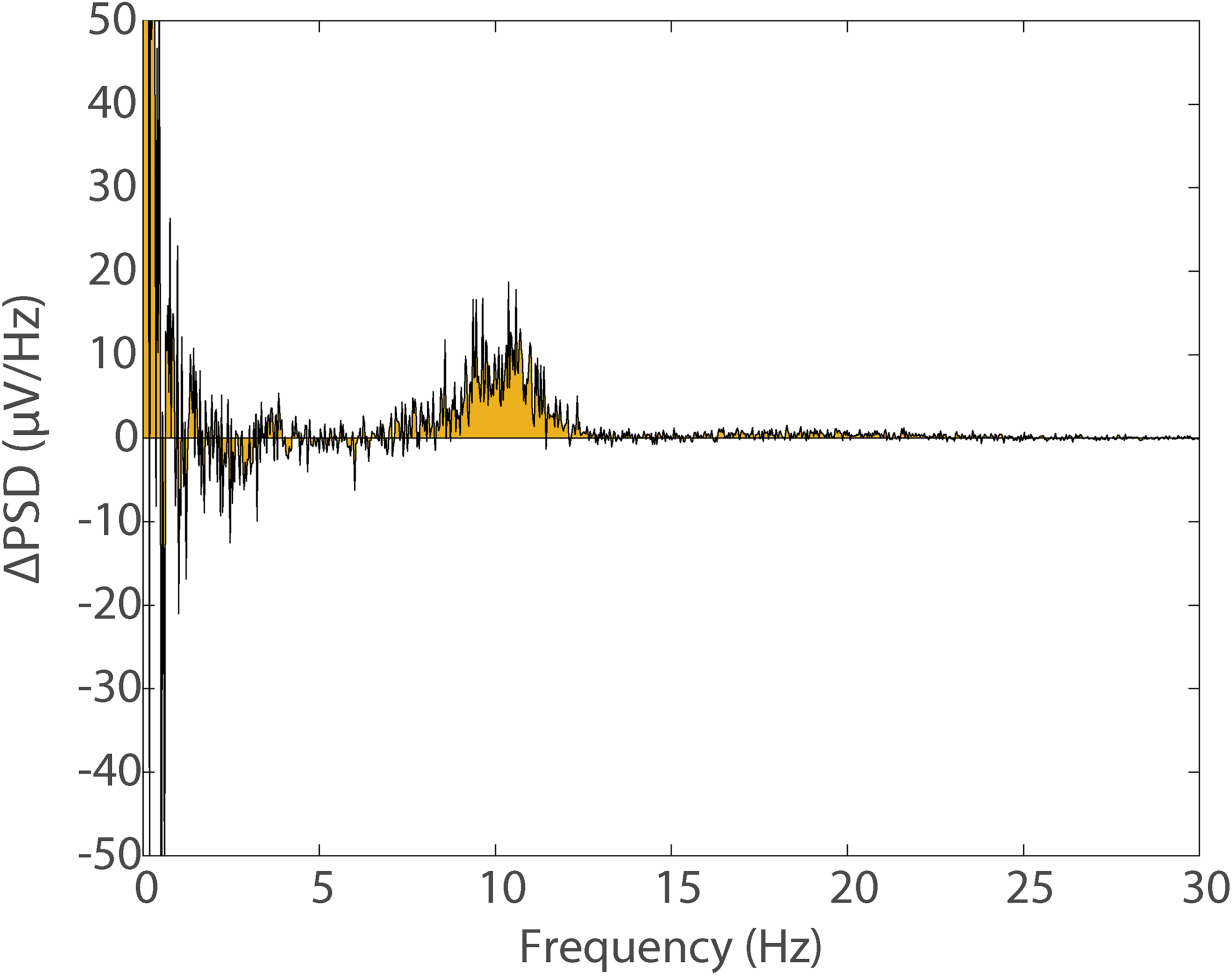
The difference in the average power spectra from electrode O1 for the eyes‐open and eyes‐closed conditions (generated from FFT's of open/closed responses), measured outside the MRI environment. Yellow shading denotes area under the spectrum to aid visualisation. This plot provides a gold standard for comparison with MA corrected data (see Figure 5) [Color figure can be viewed at http://wileyonlinelibrary.com]

Figure 4 shows an alpha signal increase between 8 and 13 Hz was induced when the subject closed their eyes. This increase was easily visible when no MAs were present in the data and provides a “gold standard” power spectrum which can be compared with the MA corrupted data after MA correction.

**Figure 5:**
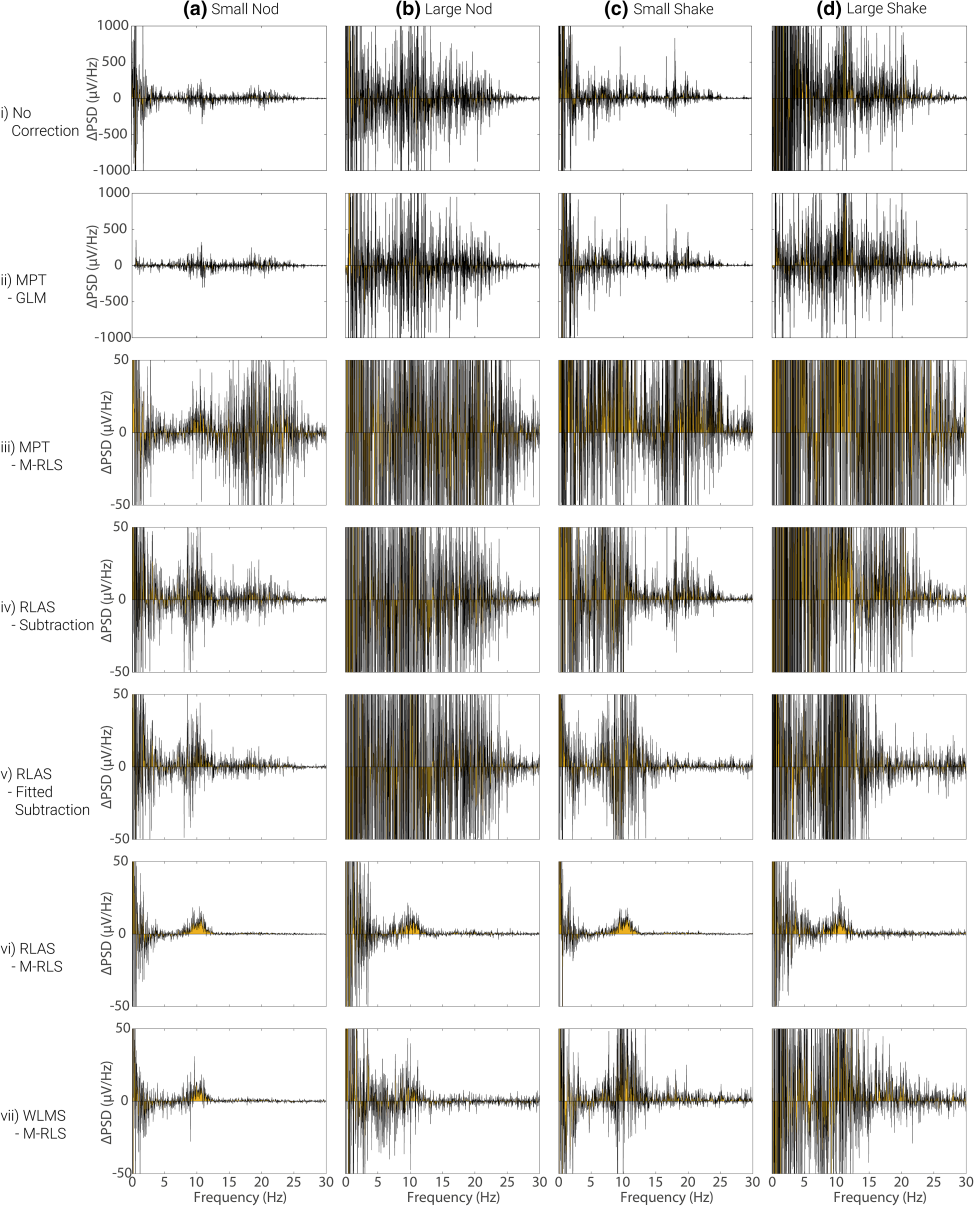
The difference in the average power spectra from electrode O1 for eyes‐open and eyes‐closed conditions (generated from FFT's of open/closed response) where MAs have been added, row i, and subsequently corrected with different methods, rows ii to vii. MA data and motion recordings used for this figure are from dataset 1. Note the different scales in the spectra plotted in rows i and ii compared with rows iii to vii and Figure 4. Yellow shading denotes the area under the spectrum to aid visualisation. See Supporting Information Figure S4 for corresponding plots for dataset 2 [Color figure can be viewed at http://wileyonlinelibrary.com]

Figure 5 shows the effect of adding the MA to the neuronal data without any correction (row i) and after each type of correction (rows ii to vii). As expected the large nod (column b) and large shake (column d) produce much greater artefacts over a broad frequency range than the corresponding smaller movements (columns a and c). Whilst MAs were largest for frequencies below 5 Hz, the artefacts at higher frequencies still dominate the neuronal signals of interest in the alpha band and surrounding frequency range for all movement types, making the neuronal alpha signal impossible to identify in the raw, MA‐corrupted data (Figure 5 row i, compared with Figure 4). Figure S3 shows the residual artefacts remaining after subtraction of the neuronal data shown in Figure 4 from the data in Figure 5.

The variation in the efficacy of the different correction methods was considerable, as revealed in Figure 5 rows ii–vii. The M‐RLS fitting approach (rows iii, vi and vii) outperformed the other methods of post‐processing correction, regardless of the method used for motion signal detection (i.e., RLAS, WLMS or MPT). The worst MA correction was provided by the MPT marker with the alpha power signal unclear after MA correction for all movement types (rows ii and iii). The best MA correction appears to be achieved by using the RLAS motion measures combined with the M‐RLS fitting algorithm (row vi). With this combination, the original alpha band signal was clearly visible after MA‐correction for the small‐amplitude head movements and there was evidence of its presence for the large amplitude head movements, especially for the nodding motion, although considerable artefact was still present. Using the WLMS data it was also possible to recover the alpha signal for the small nod movement, but not the other movement types (Figure 5, row vii). The second dataset, where larger movements were generated (Table 1) produced similar results (see, Supporting Information Figures S4 and S5).

It should be noted that even with the best correction, that is afforded by RLAS combined with M‐RLS, considerable artefact is still present in the power spectra at frequencies below 5 Hz (Figure 5 and Supporting Information Figure S4, row vi c.f. Figure 4). In addition, the MA correction appears to perform better in both datasets for head nod (Figure 5 and Supporting Information Figure S4, columns a and b), rather than head shake (Figure 5 and Supporting Information Figure S4, columns c and d) movements.

### 3.4 Qualitative assessment of the VEP data

**Figure 6:**
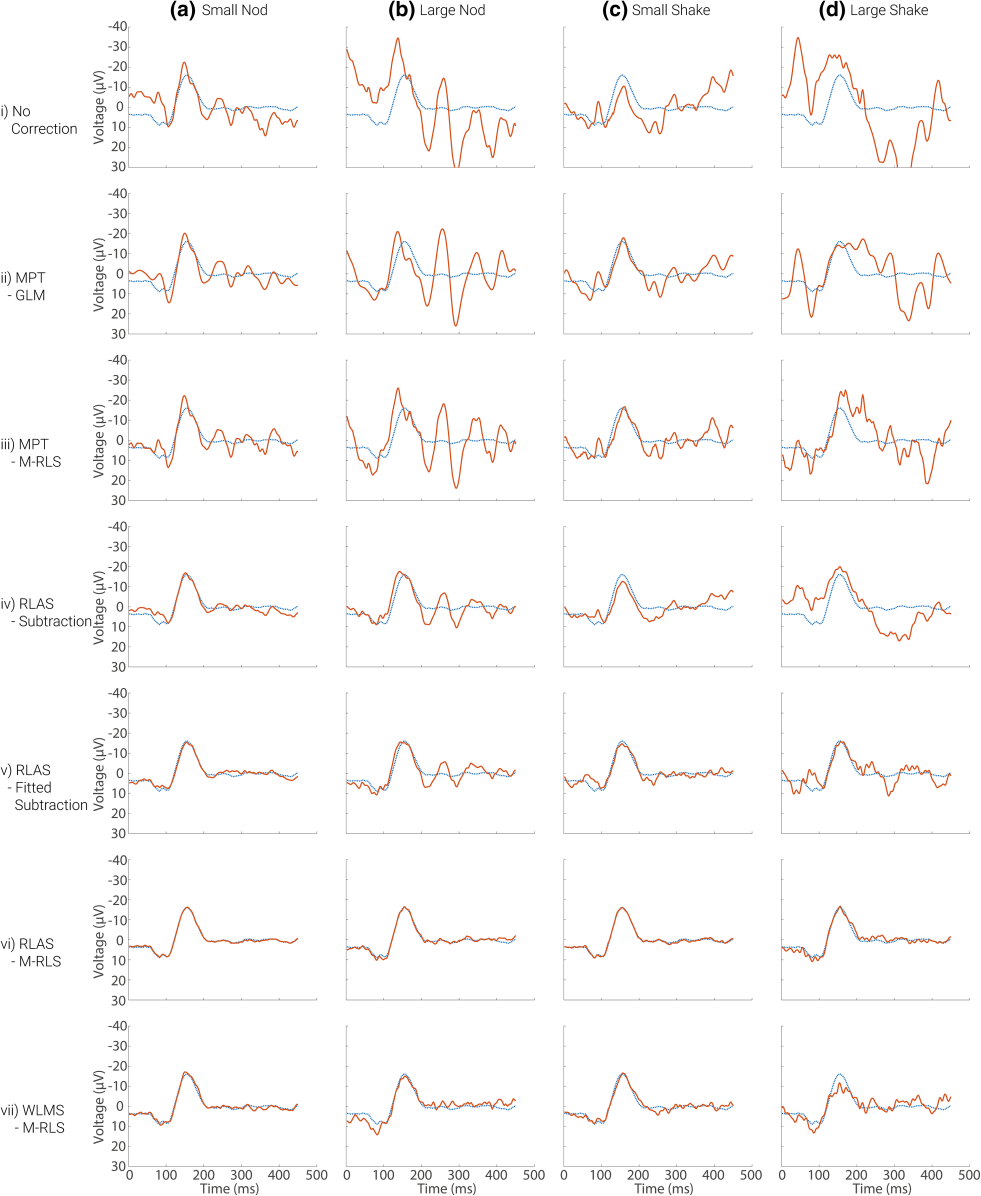
The mean VEP measured from electrode O1, averaged over 240 trials. The mean gold standard VEP is shown by the blue line with the red lines showing responses with addition of MAs from dataset 1 (row i) and after MA correction using each of the methods (rows ii to vii). Similar results for the MAs from dataset 2 are shown in Supporting Information Figure S6 [Color figure can be viewed at http://wileyonlinelibrary.com]

**Figure 7:**
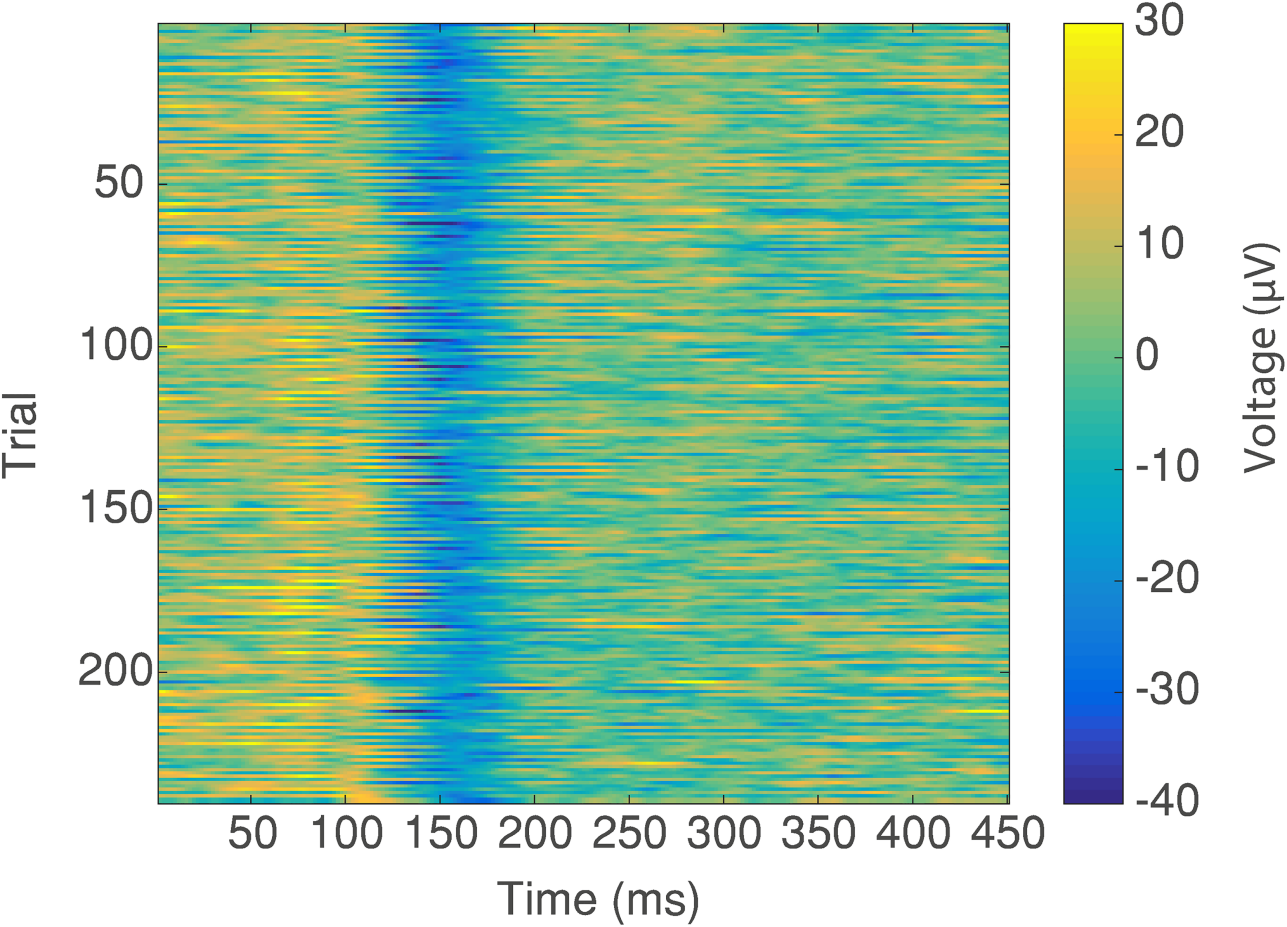
The “gold standard” neuronal VEP signals measured from electrode O1 for each individual trial (y‐axis) over the 450 ms period following stimulus onset (x‐axis). Colour illustrates the voltage measured at each time point and in each trial, with the P100 and N150 peaks clearly visible (yellow and blue strips, respectively) on the vast majority of trials [Color figure can be viewed at http://wileyonlinelibrary.com]

**Figure 8:**
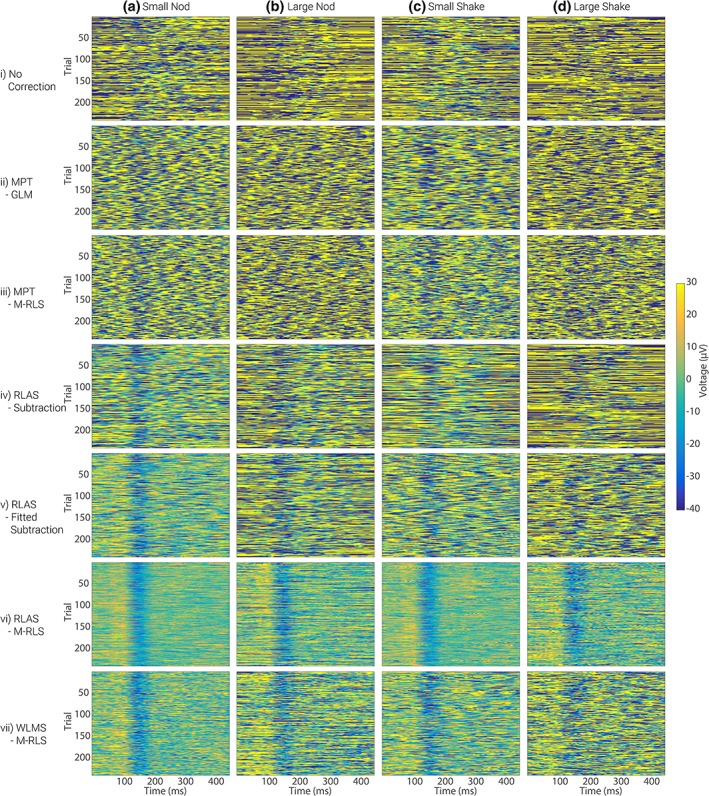
The VEP signals measured from electrode O1 for each individual trial (y‐axis) over the 450 ms period following stimulus onset (x‐axis), with the MAs from dataset 1 added (row i). Rows ii–vii show the VEP responses that are revealed after each of the MA correction methods has been applied. Colour illustrates the voltage measured at each time point and in each trial. Similar results for the MAs from dataset 2 are shown in Supporting Information Figure S7 [Color figure can be viewed at http://wileyonlinelibrary.com]

The effect of the different MA correction methods on the average VEP for the four different movements is shown in Figure 6. The blue line shows the average VEP measured from electrode O1, from the recording outside of the MRI environment (i.e., the “gold standard” response). The effect on the average VEP of adding the different MAs to the gold‐standard data is shown in Figure 6, row (i). Since the MAs were not time or phase locked to the visual stimulus presentation a considerable proportion of the MA is removed through the averaging process such that, even with no MA correction, an average VEP (averaged over 240 trials) is clearly revealed for small amplitude head movements (columns a and c). However, artefact is still clearly present despite the extensive averaging, and this dominates for the larger movements (Figure 6 row i, columns b and d). Furthermore, it is important to consider the ability to detect the true VEP amplitude on a single trial basis as this is the type of metric often used to inform the GLM used in fMRI analysis when performing EEG‐fMRI (e.g., Debener et al., 2005; Eichele et al., 2005; Mayhew, Porcaro, Ostwald, & Bagshaw, 2010). Figure 8, row i, shows that compared with the original neuronal signal, shown in Figure 7, the single trial VEPs cannot be recovered from the raw MA‐corrupted data as the yellow strip at approximately 100 ms and blue strip at approximately 150 ms (the P100 and N150) visible in Figure 7 cannot be seen in the MA‐corrupted data in Figure 8. Thus MA correction methods need to be considered for recovering VEPs, as well as oscillatory responses.

Using the MPT motion data for correction removes some of the MA (Figure 6, rows ii and iii), however, considerable residual artefact means there is still not a good correspondence between the original average VEP and the MPT MA‐corrected data. Furthermore, it is still not possible to see the single trial VEPs in the stack plots when using MPT MA correction (Figure 8, rows ii and iii). In agreement with our finding for the oscillatory responses, the best recovery of the original neuronal signal is achieved using the RLAS motion measures with the M‐RLS fitting algorithm (Figures 6 and 8, row vi). Using this method, the average VEP shows excellent correspondence with the original data for all movement types, revealing only small discrepancies compared with the original response for the larger amplitude head movements. This finding is also borne out by the single trial responses (Figure 8). The presence of the VEP in the average and single trial responses is relatively clear for the larger amplitude head movements. The correction using WLMS data with the M‐RLS fitting also provide good correspondence of the averaged VEP after MA correction for small amplitude head movements. However, greater differences using this correction approach are seen on the single trial data (Figure 8, row vii compared with Figure 7). Similar findings to these were obtained for dataset 2 in which the MAs were larger (Supporting Information Figures S4 and S5), although larger residual MAs remained after all correction methods due to the increased MAs incurred.

### 3.5 Quantitative assessment of data

**Figure 9:**
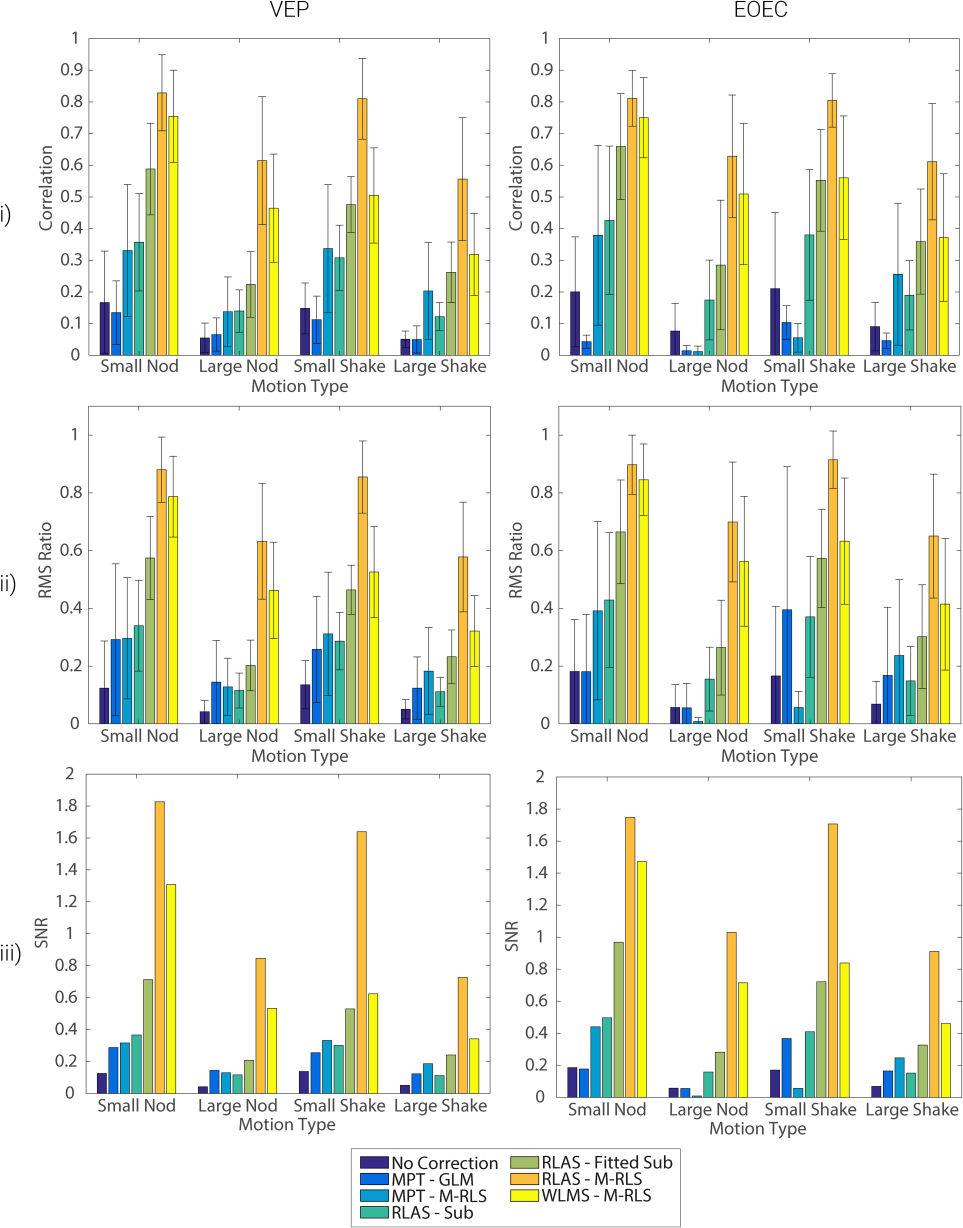
Comparison over all electrodes of the relative performance of the different methods for correcting MA from EEG data, averaged over all electrodes. Comparisons are made for the evoked (VEP), left column, and oscillatory (EOEC), right column, data. Metrics are derived for the neuronal response data combined with the MA data from dataset 1. Results with no MA correction are shown in dark blue and compared with each of the MA correction methods (see legend). Row (i) shows the results of the correlation analysis; row (ii) shows the results from the RMS ratio analysis and row (iii) shows the outcome of the SNR analysis. Bars show the mean result over all electrodes on which MA data were recorded, while error bars denote the standard deviation of these metrics over electrodes. Standard deviations of SNR are shown separately in Supporting Information Figure S9. Similar results for MA dataset 2 are shown in Supporting Information Figure S8 [Color figure can be viewed at http://wileyonlinelibrary.com]

The quantitative assessment of the relative performance of the MA correction methods is provided in Figure 9 for dataset 1 and Supporting Information Figure S8 for dataset 2. Topographical representations of the different methods’ performance measures for dataset 2 are shown in Supporting Information Figures S12–S14, along with maps of the RMS magnitude of the recorded MA (Supporting Information Figure S11). For all three metrics, a larger value illustrates better efficacy of MA correction. The first row shows the correlation of the different MA corrected responses with the original “gold standard” dataset. This clearly shows that RLAS M‐RLS provides the best motion correction for these data in terms of the correlation measure. Figure 9 indicates that this finding holds when considering all channels distributed over the head, not just the channel showing the clear occipital response to each task, as shown in Figures 5, 6, 7, 8. Interestingly the MPT correction methods showed a reduction in the correlation of the corrected signal with the original signal (light blue) compared with the non‐corrected MA corrupted data (dark blue) for some movement types, particularly for the EOEC dataset. This observation held for both MA datasets (Figure 9 and Supporting Information Figure S8) and suggests that the MA correction using the MPT in these cases has a negative effect on the EEG data quality.

The RMS ratios (Figure 9 and Supporting Information Figure S8, row ii) also show that the best performance was achieved with the RLAS M‐RLS correction. Optimal performance would result in an RMS ratio of 1 which would show the amplitude of the responses from the original data and MA corrected data were identical. The reduced RMS ratio amplitude observed with all MA correction methods tested, shows the RMS of the signal after correction was still larger than the original neuronal signal. This finding strongly suggests that residual MA remained, which is in agreement with the qualitative assessments described above. In general, all MA correction methods reduced the amplitude of the overall signal compared with no MA correction, suggesting an improvement in signal quality over all electrodes was normally achieved.

The largest difference between correction approaches was seen in the SNR metric (Figure 9 and Supporting Information Figure S8, row iii) where the RLAS M‐RLS and WLMS M‐RLS methods clearly showed large improvements compared with all other methods for all movement types. A high degree of variability in this measure over electrodes was seen for both datasets (Supporting Information Figure S9) since in the frontal electrodes the neuronal signal was very small compared with the occipital electrodes due to the nature of the visual stimuli used.

## 4 DISCUSSION

### 4.1 MA correction performance

All methods performed better (i.e., the magnitude of the residual MA was smaller) for the smaller head movements than for the larger movements. This is likely to be due primarily to the reduced magnitude of the MA induced by these smaller movements. Although, it is also likely that the large MAs are not corrected as well by fitting procedures, such as M‐RLS, because the artefact morphology changes faster (more rapid movement through the static magnetic field) and as a result the weights of the fitting do not adapt sufficiently quickly, as previously discussed (Jorge et al., 2015). For these large amplitude head movements our results show residual MA is present in the EEG data regardless of which MA correction method employed. Therefore, the reduced performance of the MA correction cannot be solely due to the faster changing artefacts. Although the MA correction is not perfect for larger MAs, by acquiring motion data, separate from the EEG data containing the neuronal activity, it should be possible to visually inspect the motion and EEG data together to identify when residual MAs are present, and thus to decide which data segments must be excluded even after MA correction. Thus, such monitoring will provide a method by which to overcome limitations faced in previous simultaneous EEG‐fMRI studies where MAs were present, for example, Jansen et al. (2012); but effected data could not be removed due to a lack of information regarding the temporal occurrence of the MA.

Qualitatively, data recorded from electrode O1 showed that MA correction methods performed best for the artefact induced by a head nod. When considering the quantitative analyses for the small amplitude head movements, the movements were very similar in amplitude for the nod and shake in dataset 1, which is borne out by the similar metrics calculated for the two movement types before any correction (Figure 9, dark blue bars). The correlation and RMS ratio also show similar performance for these data when the best correction method, RLAS M‐RLS, was used. However, an increase in the SNR measure for the nod relative to the head shake was observed, suggesting improved MA correction for a head nod (Figure 9, row iii, orange bars). When considering dataset 2 where the small amplitude head shake was considerably smaller than the nod (RMS difference = 0.6 mm), the best MA correction method (RLAS M‐RLS) showed worse performance for all three metrics for the shake than the nod motion (Supporting Information Figure S8, orange bars). A similar pattern is seen for the large movements in dataset 1 (Figure 9), but the discrepancy in the size of head movement for the large amplitude nod and shake movements of dataset 2 (Table 1) means that the correction of the MA for head shake was found to be superior (Supporting Information Figure S8). Together these results suggest a slightly improved performance in correcting the artefact induced by a head nod than a head shake. This movement type is likely to be the most common form of gross head movement generating MAs in EEG‐fMRI studies as it is the easiest movement for a subject to make when the head is inside the RF head coil. Furthermore a large component of the pulse artefact is believed to be caused by a nodding motion (Yan et al., 2010), which may explain the considerable success of all the tested methods at removing the pulse artefact (Jorge et al., 2015; LeVan et al., 2013; Masterton et al., 2007).

The difference in performance of the MA correction for a head nod and shake is interesting as analysis of a simple model of the head as a sphere with the EEG leads following lines of longitude suggests that head shake should induce no MA, as the flux linked by the effective wire loops formed by the leads and head does not change (Yan et al., 2010). Although this analysis is based on a very simplistic model, which does not correspond to more complex wire paths in a real EEG cap, it may suggest that a greater proportion of the MA is induced in the leads, rather than the cap and head, for a head shake than a head nod. If this is the case, the RLAS M‐RLS system may outperform other methods because the starquad cable used in the construction of the cap ensures identical artefacts are induced on the reference layer wires as those on the scalp layer wires. Related effects may explain to some extent the relatively poor performance of the MPT marker method: measurements of the movement of a single marker attached to the head do not capture movements of the EEG leads that are not fully correlated with the head movement. From our analyses thus far, it is unclear as to whether the superior performance of the RLAS M‐RLS over the WLMS M‐RLS method for MA correction (Figure 9 and Supporting Information Figure S8) is due to: (i) the number of MA detection channels used (9 in the case of RLAS and only 4 in the case of WLMS); or (ii) the RLAS system better capturing the MA induced (either through the reference layer better mimicking the scalp or due to the starquad cable better capturing the MAs induced in the leads linking the electrodes and amplifier) than is possible with the four wires of the WLMS system.

**Figure 10:**
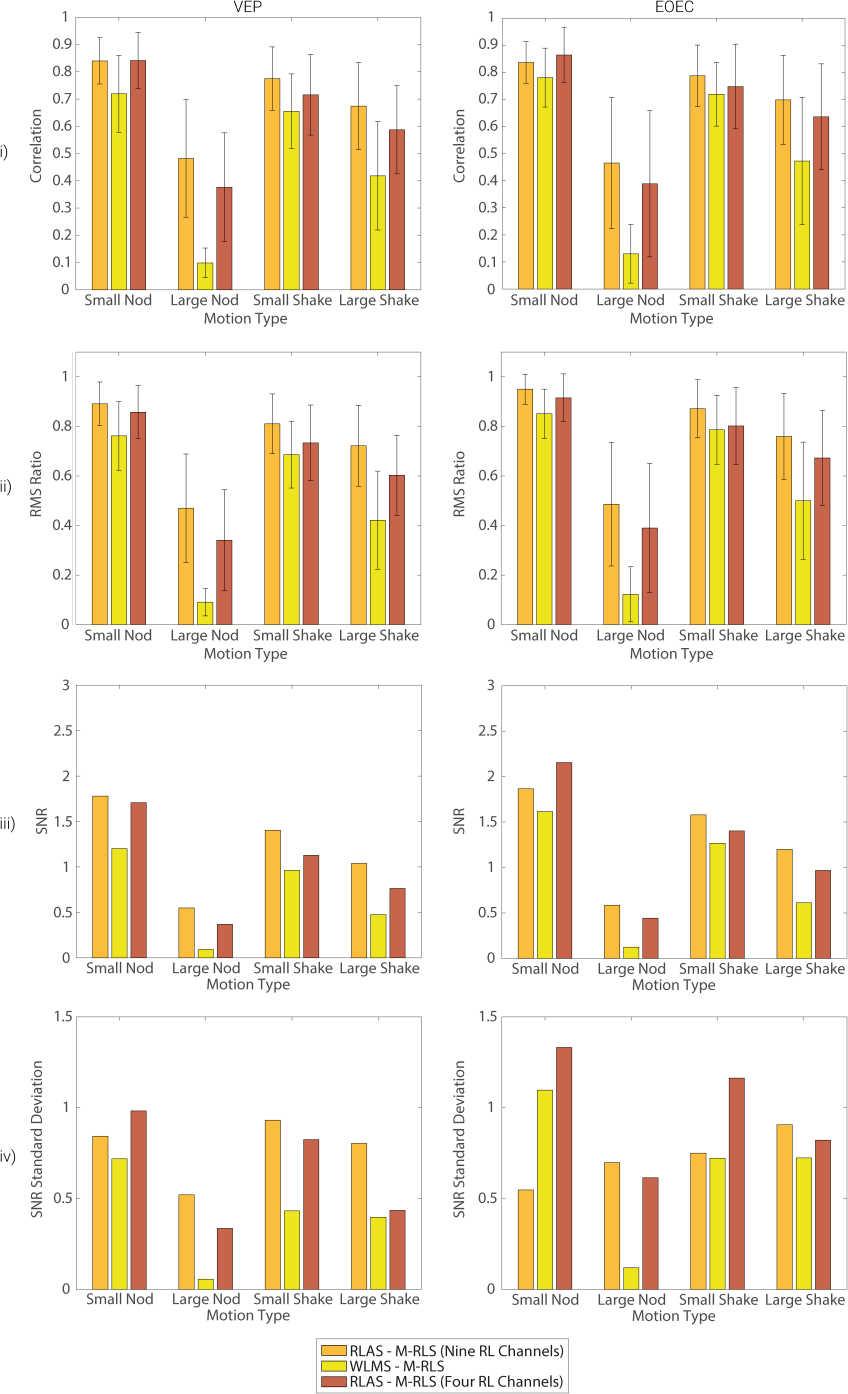
Comparison over all electrodes of the relative performance of RLAS M‐RLS using all available reference layer channels (9), WLMS M‐RLS and RLAS M‐RLS using selected reference layer channels (4: Fc5, Fc6, Cp5 and Cp6) for correcting MA from EEG data. Comparisons are made for the evoked (VEP), left column, and oscillatory (eyes open/closed [EOEC]), right column, neuronal response data combined with the MA data from dataset 2. Row (i) shows the results from the correlation analysis, row (ii) the results from the RMS ratio analysis and row (iii) the outcome of the SNR analysis. Bars show the mean result over all electrodes on which MA data were recorded, while error bars denote the standard deviation of these metrics over electrodes. The standard deviation of the SNR was large due to the lack of neuronal signal on frontal electrodes and is, therefore, shown in a separate plot (row iv) [Color figure can be viewed at http://wileyonlinelibrary.com]

To test which of these factors explained the differences observed between methods (Figure 9 and Supporting Information Figure S8 orange: RLAS M‐RLS; yellow: WLMS M‐RLS) the RLAS M‐RLS MA correction was also performed using only 4 reference channels. The RLAS channels closest to the WLMS channels were chosen (Fc5, Fc6, Cp5 and Cp6). This additional analysis was only carried out on dataset 2, since recordings from all of these channels were not available in dataset 1. The results are shown in Figure 10. Crucially, the reduced channel RLAS M‐RLS fit regardless of number of reference channels outperformed the WLMS method over all EEG channels for all movement and data types and for all metrics of MA correction performance (Figure 10). This result suggests that the superior performance of RLAS M‐RLS was not solely due to the number of channels of the RLAS system. It appears that the geometry/conductance of the reference layer or the use of the starquad cable to match the MAs induced in the wires emanating from the scalp and reference layer electrodes also plays an important role and warrants further development (see “*Future of motion monitoring for MA correction”* section below).

Generally, the RLAS M‐RLS fitting performed similarly for most movement types when using 4 channels compared with 9 channels. Surprisingly, for the small amplitude head nod the reduced channel RLAS M‐RLS system outperformed the full 9 channel MA correction. On visual inspection of the corrected data it appears that this difference in performance was driven by too large a weighting given to channels over the occipital cortex, which were relatively insensitive to the head nod (with a right–left topography (Yan et al., 2010)). However, these occipital channels contained some high frequency artefact components which drove their weightings for the MA correction and appeared to reduce the weightings of the channels used in the reduced channel system, resulting in the difference in performance observed. Therefore, if head nods were the only movement then a reduced channel RLAS reference layer system may be beneficial. However, head shakes will induce larger artefacts over frontal and occipital electrodes (anterior–posterior MA topography) and therefore distributing the reference layer electrodes over the scalp surface is likely to be advantageous for overall correction of MA due to types of movements.

### 4.2 Retaining neuronal signal

When any fitting procedure is used to remove a noise source (in this case the MA) there is always the possibility that overfitting may occur, particularly when the underlying neuronal signal and the noise source are correlated over the timescale that the fitting is performed. Such overfitting would be particularly problematic in the case of simultaneous EEG‐fMRI where single trial features of the EEG response, such as ERP amplitude (e.g., Debener et al., 2005; Eichele et al., 2005; Mayhew, Porcaro, et al., 2010) or variability in oscillatory power (e.g., Goldman et al., 2002; Laufs et al., 2003; Mayhew, Porcaro, et al., 2010, 2013; Mullinger et al., 2013, 2014; Scheeringa et al., 2016) are commonly used to inform modelling of the fMRI signals. If amplitudes are artificially reduced non‐systematically (e.g., during periods with no movement, where the lack of MA means the fitting is biased to neuronal signals, but not during periods of subject movement) measurement of single trial amplitudes would be inaccurate, potentially leading to incorrect inferences being drawn from EEG‐fMRI studies.

Previous studies, in which motion metrics were fitted to EEG scalp data, have shown that neuronal signals are recoverable (Chowdhury et al., 2014; Jorge et al., 2015; LeVan et al., 2013; Masterton et al., 2007; Maziero et al., 2016; Steyrl et al., 2017). However, the ability to obtain the true underlying signal and the accompanying trial‐by‐trial variations of these responses could not be assessed in these studies, since the precise form of the underlying neuronal signals was not known (since the neuronal and MA signals were acquired in the same acquisition). Masterton et al. (2007), characterised the ability to recover a simulated 10 Hz oscillatory signal and showed that their wire loop motion detection method combined with the M‐RLS fitting was able to recover this signal. However, a pure 10 Hz oscillation only roughly approximates true neuronal activity, which contains features over a broad frequency range as well as ERPs, both of which can have very similar temporal profiles to short MAs. Thus, the overfitting of motion metrics to the MA corrupted EEG neuronal data is conceptually likely.

Our results suggest that none of the tested MA correction methods that exploited data fitting steps resulted in significant removal of neuronal signals. This is reflected by the fact that the calculated RMS ratio never exceeded a value of 1 (Figure 9 and Supporting Information Figure S8, row ii). Perfect correction of the MA would result in an RMS ratio of 1, with a value greater than 1 meaning that there was a reduced signal amplitude after correction compared with the “gold standard” neuronal signal, providing strong indication of over‐fitting. An RMS ratio > 1 was not observed for either the evoked or oscillatory responses (Figure 9 and Supporting Information Figure S8). Although removal of neuronal signal (i.e., over‐fitting) whilst MA remained could result in the RMS ratio <1 (the RMS ratio we observed), the qualitative analysis performed does not support this scenario as the source of our findings. The average evoked potentials after MA correction either closely followed the gold standard signal in terms of amplitude of the response or were generally larger than the gold standard signal (Figure 6 and Supporting Information Figure S6), indicating no over‐fitting of the neuronal signal. The only exception to this is the WLMS M‐RLS correction of a large amplitude head shake data (Figure 6, row vii). However, as all other uses of M‐RLS with the different motion metrics did not result in a smaller amplitude signal, we believe this result is unlikely an effect of overfitting, and more likely due to residual MA causing partial cancellation of the VEP.

As discussed, the trial‐by‐trial variability of ERPs is often measured during simultaneous fMRI. Such variability is evident in Figure 8 and Supporting Information Figure S7 and there appears to be no systematic difference (i.e., reduction/increase) in the VEPs after MA correction compared with the gold standard responses (Figure 7). When considering, the best MA correction method tested (RLAS M‐RLS), the difference between the MA‐corrected data and the gold standard is minimal especially for the case of the small movements (see Supporting Information Figure S10). The lack of any structure across trials in the residual signal shown in Supporting Information Figure S10, indicated that overfitting was not a problem in this best‐case scenario and that the remaining differences between the MA corrected data and the gold‐standard data (shown in Supporting Information Figure S10) is residual MA and noise in the EEG data. Inspection of the qualitative results for the oscillatory responses reveals a similar pattern, with no obvious decreases in the alpha band responses after MA correction (Figure 5 and Supporting Information Figure S3) compared with the gold standard (Figure 4).

Therefore, from these investigations we conclude that over‐fitting of the data was not a problem for the motion metrics and fitting algorithms tested here. This is somewhat surprising given the large number of weightings involved in some of the M‐RLS filters, where the number of weights is given by (2 × *l* + 1) × *m* (where *l* is the filter length and *m* is the number of motion channels). In the case of the RLAS M‐RLS filter this amounts to a total of 248 weightings (for 8 channel system) applied at each time point of the dataset. A filter length of 15 and down‐sampling factor of 3, as used here, results in filter length of 0.186 s ([({2 × *l*} + 1) × *dsf*/*f*], where *dsf* = down‐sampling factor, and *f* = sampling frequency of EEG data) which is iteratively applied to each sample point of the EEG dataset. Such a filter might be expected to result in overfitting due to its short duration. In addition, the adaptability factor could also result in overfitting if the weights are allowed to change too rapidly and therefore care must be taken in choosing this and how it interacts with the filter length (Supporting Information Figure S1). While no over‐fitting was observed here, this does not guarantee that over‐fitting will not occur if different parameters are used in the fitting procedure, or an increase number of motion channels are used, see “*Future of motion monitoring for MA correction*” section.

### 4.3 Limitations of study

Since the purpose of this study was try and recover a known neuronal signal related to a task, the MA and neuronal signals were entirely recorded independently. However, in true EEG‐fMRI data it is possible that some neuronal signals may be time‐locked to the MAs, especially neuronal signals that are related to the planning and execution of movement (Jansen et al., 2012). Here, we did not assess the ability of the different motion correction methods to recover neuronal signals related to motion in the presence of correlated MAs. This issue might be addressed in future work by analysing signals produced by recording such neuronal signals outside the scanner and then overlaying temporally correlated MAs recorded from a phantom. In general however, unless the investigation of neuronal activity due to movement is the goal of a study, it may not be a problem if such movement‐related neuronal activity is removed during any MA correction procedure.

It is well known that head movement also produces changes in the magnitudes and morphology of GA due to changes in head position with respect to the applied gradients (Yan et al., 2009; Mullinger et al., 2011) and GA correction methods have been shown to be applicable to data affected by movements of the extent considered here (Chowdhury et al., 2014; Moosmann et al., 2009). Significant changes in head angulation also produce changes in the form of the pulse artefact (Yan et al., 2010). Since the recordings of neuronal signals used here were made outside the scanner and no gradient waveforms were applied while the measurements were made on the phantom inside the scanner, we cannot assess the effect of movements on the GA and PA. Of the methods for correcting MAs that were assessed here, only RLAS (Chowdhury et al., 2014) is designed specifically also to remove GA and PA, but further work is needed to assess the performance of the RLAS M‐RLS approach (that gave the best reduction of MAs) in attenuating these other artefacts. It is likely that information from the wire loops and MPT recordings could also be used to inform the process of GA and PA reduction – for example, by indicating when movement is sufficient to require the generation of new templates for average artefact subtraction – and further work in this area is also required if the full benefits of EEG‐fMRI are to be realised.

### 4.4 Future of motion monitoring for MA correction

The lack of overfitting observed here may not be the case if a larger number of motion metrics are recorded. This might be a relevant factor when a larger number of EEG reference layer channels are included in a full RLAS system and use of the RLAS M‐RLS approach would require further investigation in such a setup. Furthermore, given the effect of the reduction in channels when using the RLAS system in combination with M‐RLS fitting (Figure 10), the efficacy of MA correction may not be increased by adding a larger number of reference layer channels.

Users must also consider that the optimal parameters used here for M‐RLS may not be optimal if the motion data is acquired with a different sampling frequency or is subjected to filtering that is different to that used here. For example, the down‐sampling factor of 3, which we found to be optimal (Figure 3) is likely to produce the best results as it effectively reduces the maximum frequency present in the data to approximately 83 Hz (sampling rate [500]/down‐sampling factor [3] /2 [3]). However, as the motion data were also frequency filtered to 80 Hz in this study, no information is lost for the purpose of M‐RLS. Therefore, the motion channels still contain all of the low frequency MA signal, but have had the high frequency signals, (which here were primarily white noise, but which could be gradient artefact in true simultaneous EEG‐fMRI recordings) removed.

Some consideration must also be given to the computation time required for fitting using M‐RLS to be performed. This particularly important for studies that require real‐time MA correction, for example to provide neural feedback to the subject performing a task. The time for the M‐RLS fitting procedure increased by a factor of *m* ^3^ (where *m* = number of motion channels), using the computer programmes implemented in this study (time dependence on m was determined from experimentally measuring computing time for different m values; for example, it took 100 s to process a 60 s dataset with 9 motion channels). This time factor was therefore a considerable hindrance for fitting the MPT data using M‐RLS, where 18 motion metrics were used. However, it should be possible to significantly reduce the processing time for MA correction through streamlining the implementation of the M‐RLS algorithm. Two approaches which could be combined, are the use of a lower level computing language for example, C++ (Masterton et al., 2007) (rather than MATLAB used here) for implementation of the algorithm and to exploit the benefits of general purpose graphical processing units (GPGPUs) in parallelising the processing. Such implementations were beyond the scope of this investigation and require work in the future to test feasibility.

In thinking about the implementation of MA correction it is also important to consider the experimental practicalities. The MPT‐marker approach is arguably the easiest to implement, but it appears to perform considerably worse than the other methods for correcting MA and therefore is unlikely to become the method of choice. WLMS as implemented here (and in Jorge et al., 2015)) is more practical than RLAS, or the originally proposed wire loops (Masterton et al., 2007) to set up, as a standard EEG cap can be used with very little modification and minimal additional hardware. While this method does require the loss of a few EEG channels (4 in the case tested here) for monitoring brain activity this is a relatively small proportion of the channels available (commonly 64 for standard EEG‐fMRI). At the moment therefore, given the lack of commercial availability of a true RLAS system and the slightly inferior performance of WLMS M‐RLS compared with RLAS M‐RLS, WLMS may currently be the method of choice for recording MA to use in MA correction. However, given the superior performance of RLAS M‐RLS a more user‐friendly adaptation of this set‐up should be developed. As mentioned previously it may be the performance of the solid reference layer which more accurately characterises the MA or it may be the presence of the starquad cable in capturing MA from the leads that is the crucial aspect of the RLAS system. It is clear therefore that to provide the best possible MA correction, further investigation is required.

## 5 CONCLUSIONS

Here, we have provided a quantitative comparison of the relative merits of different, previously proposed, methods for correcting motion artefacts induced in EEG data during simultaneous fMRI. Head motion is known to induce large artefacts in EEG data during simultaneous fMRI therefore finding the best possible method to remove the MAs is important. We assessed the relative performance of different MA correction methods by simultaneously acquiring motion information with three methods [RLAS (Chowdhury et al., 2014), MPT markers (Maziero et al., 2016) and WLMS (Jorge et al., 2015)] along with EEG data. The EEG data were acquired on a realistic head phantom such that only MAs and other (primarily white) noise were recorded. These EEG data were combined with neuronal EEG data acquired on a human subject outside of the MRI environment. The MAs were then corrected using motion information collected from each of the different methods in conjunction with number of previously described analysis pipelines (Chowdhury et al., 2014; Masterton et al., 2007; Maziero et al., 2016; Spencer et al., 2018). We showed that the MA was best corrected using the RLAS motion information combined with a multichannel recursive least squares (M‐RLS) fitting algorithm. All methods retained the neuronal signal of interest, but for several of the methods the MA was not removed sufficiently to allow accurate detection of the underlying neuronal signal.

## Supporting information


**Figure S1:** The effect of the filter length and adaptability factor (λ) on **A:** the correlation between the original (gold standard, neuronal) and corrected signals and **B:** the ratio of the RMS amplitudes of the original and corrected signal. These plots show the average of each metric over all EEG channels using 2 mins 20 secs of neuronal data (from VEP paradigm) with data from small amplitude head nods MA added and subsequently corrected, akin to Figure 3.
**Figure S2:** The change in weights as a function of time from each of the reference layer channels used to correct the O1 scalp channel for the small nod of dataset 1. To aid with visualising changes over time the de‐meaned weights are also shown.
**Figure S3:** The artefacts remaining after applying each correction method to eyes‐open‐ eyes‐closed (EOEC) data. The difference in the average power spectra from electrode O1 for eyes‐open and eyes‐closed conditions (generated from FFT's of open/closed response) where MAs have been added, row i, and subsequently corrected with different methods, rows ii‐vii. In contrast to Fig. 5, here the gold‐standard EOEC data (Fig. 4) has been subtracted, so only residual artefacts remain. MA data and motion recordings used for this figure are from dataset 1. Note the different scales in the spectra plotted across panels. Yellow shading denotes the area under the spectrum to aid visualisation.
**Figure S4:** The difference in the average power spectra from electrode O1 for eyes‐ open and eyes‐closed conditions (generated from FFT's of open/closed response) where MAs have been added, row i, and subsequently corrected with different methods, rows ii‐vii. MA data and motion recordings used for this figure are from dataset 2. Note the different scales in the spectra plotted across panels, and compared with Figure 4. Yellow shading denotes the area under the spectrum to aid visualisation.
**Figure S5:** The artefacts remaining after applying each correction method to eyes‐open‐ eyes‐closed (EOEC) data. The difference in the average power spectra from electrode O1 for eyes‐open and eyes‐closed conditions (generated from FFT's of open/closed response) where MAs have been added, row i, and subsequently corrected with different methods, rows ii‐vii. In contrast to Fig. S4, here the gold‐standard EOEC data (Fig. 4) has been subtracted, so only residual artefacts remain. MA data and motion recordings used for this figure are from dataset 2. Note the different scales in the spectra plotted across panels. Yellow shading denotes the area under the spectrum to aid visualisation.
**Figure S6:** The mean VEP measured from electrode O1, averaged over 240 trials. The mean gold standard VEP is shown by the blue line with the red lines showing responses with addition of MAs from dataset 2 (row i) and after MA correction using each of the methods (rows ii‐vii).
**Figure S7:** The VEP signals measured from electrode O1 for each individual trial (y‐axis) over the 450 ms period following stimulus onset (x‐axis), with the MAs from dataset 2 added (row i). Rows ii‐vii: show the responses that are revealed after each of the MA correction methods tested. Colour illustrates the voltage measured at each time point and in each trial.
**Figure S8:** Comparison over all electrodes of the relative performance of the different methods for correcting MA from EEG data, averaged over all electrodes. Comparisons are made for the evoked (VEP), left column, and oscillatory (eyes open/closed [EOEC]), right column, data. Metrics are derived for the neuronal response data combined with the MA data from dataset 2. Results with no MA correction are shown in dark blue and compared with each of the MA correction methods (see legend). Row i) shows the results from the correlation analysis, Row ii) shows the results from the RMS ratio analysis and Row iii) shows the outcome of the SNR analysis. Bars show the mean result over all electrodes on which MA data were recorded, whilst error bars denote the standard deviation of these metrics over electrodes. Standard deviations of SNR are shown separately, see Fig S6.
**Figure S9:** Standard deviation of the SNR metrics across channels shown in Figures 9 and S5 for dataset 1 (row i) and dataset 2 (row ii). The large standard deviations show the large variability in SNR across the channels due to the neuronal signal to the visual stimulus being much larger in the occipital electrodes, as expected.
**Figure S10:** Stack plots (as shown in Figures 7, 8 and S4) of the difference in the EEG data corrected for MA with the RLAS‐M‐RLS method (best performing method, row vi of Figs 8 and S4) and the original EEG data (i.e. the gold standard, Fig 7) for each of the movement types (columns) and dataset 1/2, (row i/ii respectively).
**Figure S11:** The topography of the RMS of the MA recorded on the scalp electrodes for dataset 2.
**Figure S12:** The topography of the correlation of the corrected signal with the gold standard for each MA correction method applied to dataset 2.
**Figure S13:** The topography of the RMS ratio of the corrected signal for each MA correction method applied to dataset 2.
**Figure S14:** The topography of the SNR of the corrected signal for each MA correction method applied to dataset 2.
