# Supplementary material for: Exploring the relative efficacy of motion artefact correction techniques for EEG data acquired during simultaneous fMRI
Source: Hum Brain Mapp. 2018 Oct 19;40(2):578–96. doi: 10.1002/hbm.24396 (PMC6492138; doi:10.1002/hbm.24396)
Supplement: Supplementary file 1 — Figure S1: The effect of the filter length and adaptability factor (λ) on A: the correlation between the original (gold standard, neuronal) and corrected signals and B: the ratio of the RMS amplitudes of the original and corrected signal. These plots show the average of each metric over all EEG channels using 2 mins 20 secs of neuronal data (from VEP paradigm) with data from small amplitude head nods MA added and subsequently corrected, akin to Figure 3. Figure S2: The change in weights as a function of time from each of the reference layer channels used to correct the O1 scalp channel for the small nod of dataset 1. To aid with visualising changes over time the de‐meaned weights are also shown. Figure S3: The artefacts remaining after applying each correction method to eyes‐open‐ eyes‐closed (EOEC) data. The difference in the average power spectra from electrode O1 for eyes‐open and eyes‐closed conditions (generated from FFT's of open/closed response) where MAs have been added, row i, and subsequently corrected with different methods, rows ii‐vii. In contrast to Fig. 5, here the gold‐standard EOEC data (Fig. 4) has been subtracted, so only residual artefacts remain. MA data and motion recordings used for this figure are from dataset 1. Note the different scales in the spectra plotted across panels. Yellow shading denotes the area under the spectrum to aid visualisation. Figure S4: The difference in the average power spectra from electrode O1 for eyes‐ open and eyes‐closed conditions (generated from FFT's of open/closed response) where MAs have been added, row i, and subsequently corrected with different methods, rows ii‐vii. MA data and motion recordings used for this figure are from dataset 2. Note the different scales in the spectra plotted across panels, and compared with Figure 4. Yellow shading denotes the area under the spectrum to aid visualisation. Figure S5: The artefacts remaining after applying each correction method to eyes‐open‐ eyes‐closed (EOEC) data [file HBM-40-578-s001.pdf]

Supporting Information

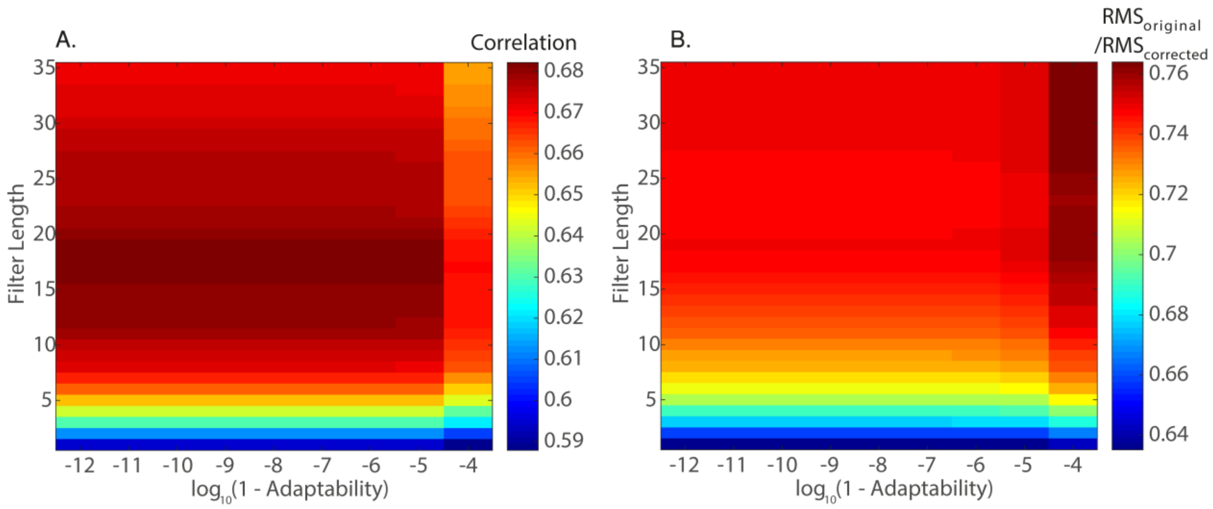

Figure S1: *The effect of the filter length and adaptability factor (λ) on* **A:** *the correlation between the original (gold standard, neuronal) and corrected signals and* **B:** *the ratio of the RMS amplitudes of the original and corrected signal. These plots show the average of each metric over all EEG channels using 2 mins 20 secs of neuronal data (from VEP paradigm) with data from small amplitude head nods MA added and subsequently corrected, akin to Figure 3.*

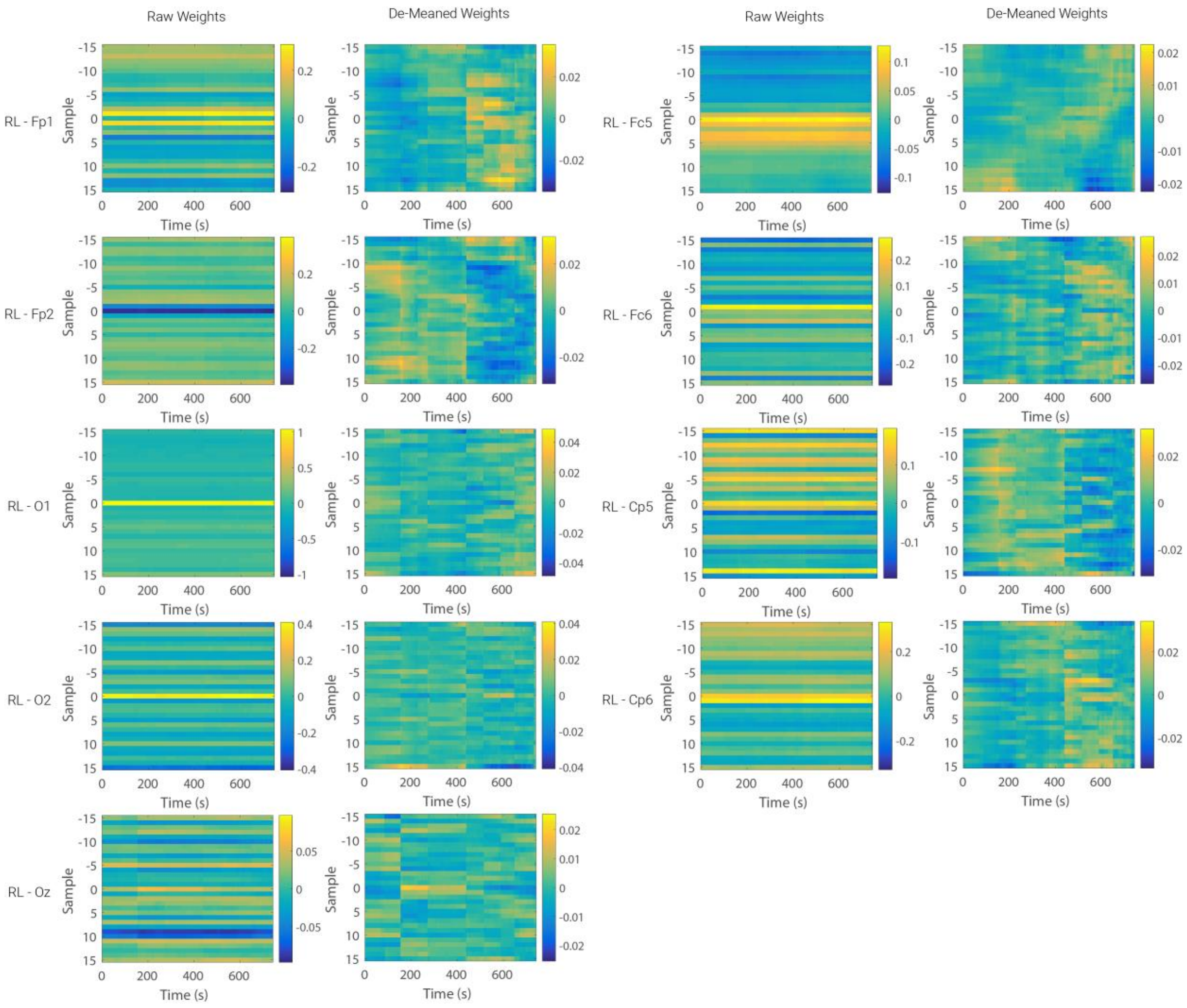

Figure S2: *The change in weights as a function of time from each of the reference layer channels used to correct the O1 scalp channel for the small nod of dataset 1. To aid with visualising changes over time the de-meaned weights are also shown.*

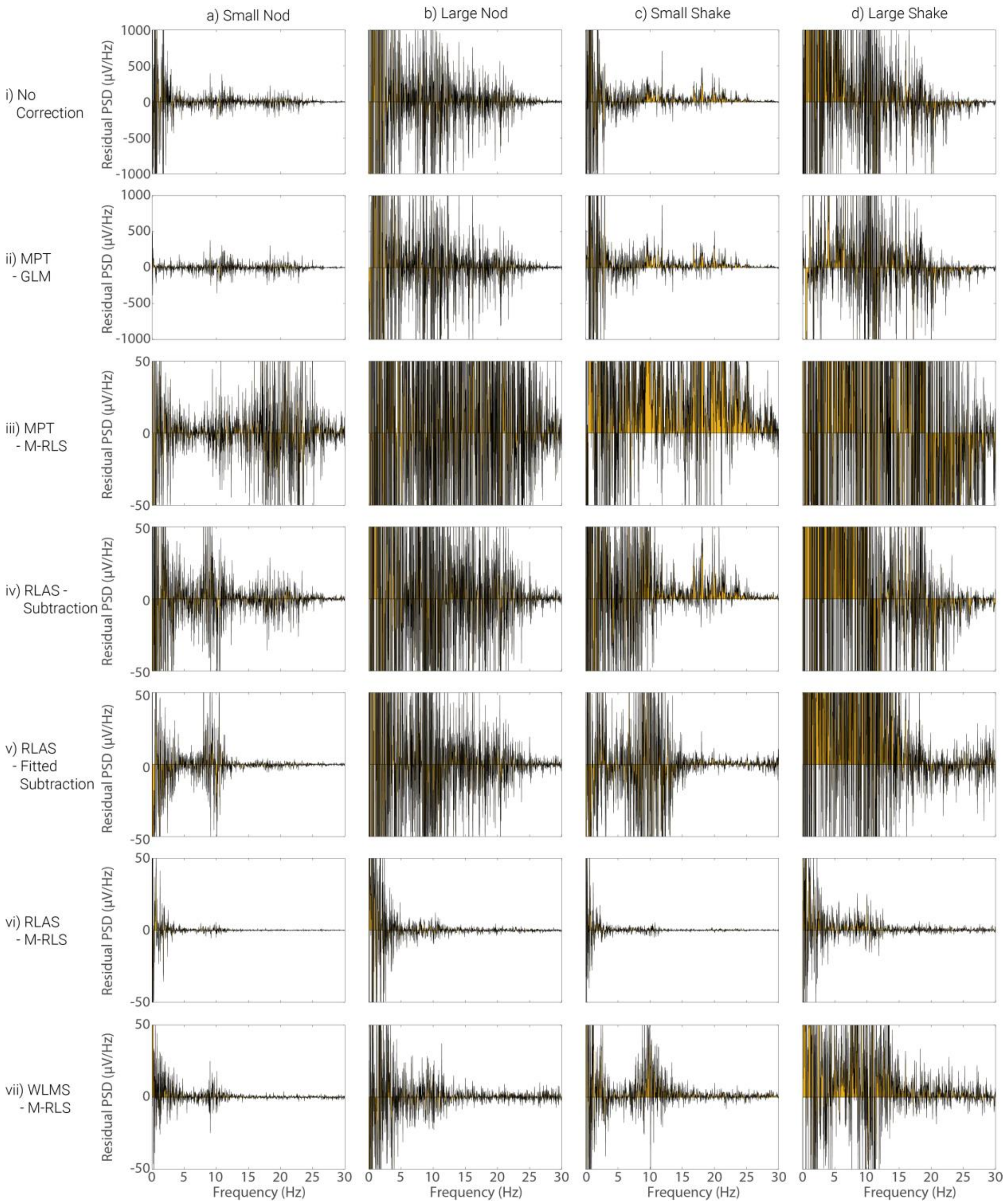

Figure S3: *The artefacts remaining after applying each correction method to eyes-open-eyes-closed (EOEC) data. The difference in the average power spectra from electrode O1 for eyes-open and eyes-closed conditions (generated from FFT's of open/closed response) where MAs have been added, row i, and subsequently corrected with different methods, rows ii-vii. In contrast to Fig. 5, here the gold-standard EOEC data (Fig. 4) has been subtracted, so only residual artefacts remain. MA data and motion recordings used for this figure are from dataset 1. Note the different scales in the*

spectra plotted across panels. Yellow shading denotes the area under the spectrum to aid visualisation.

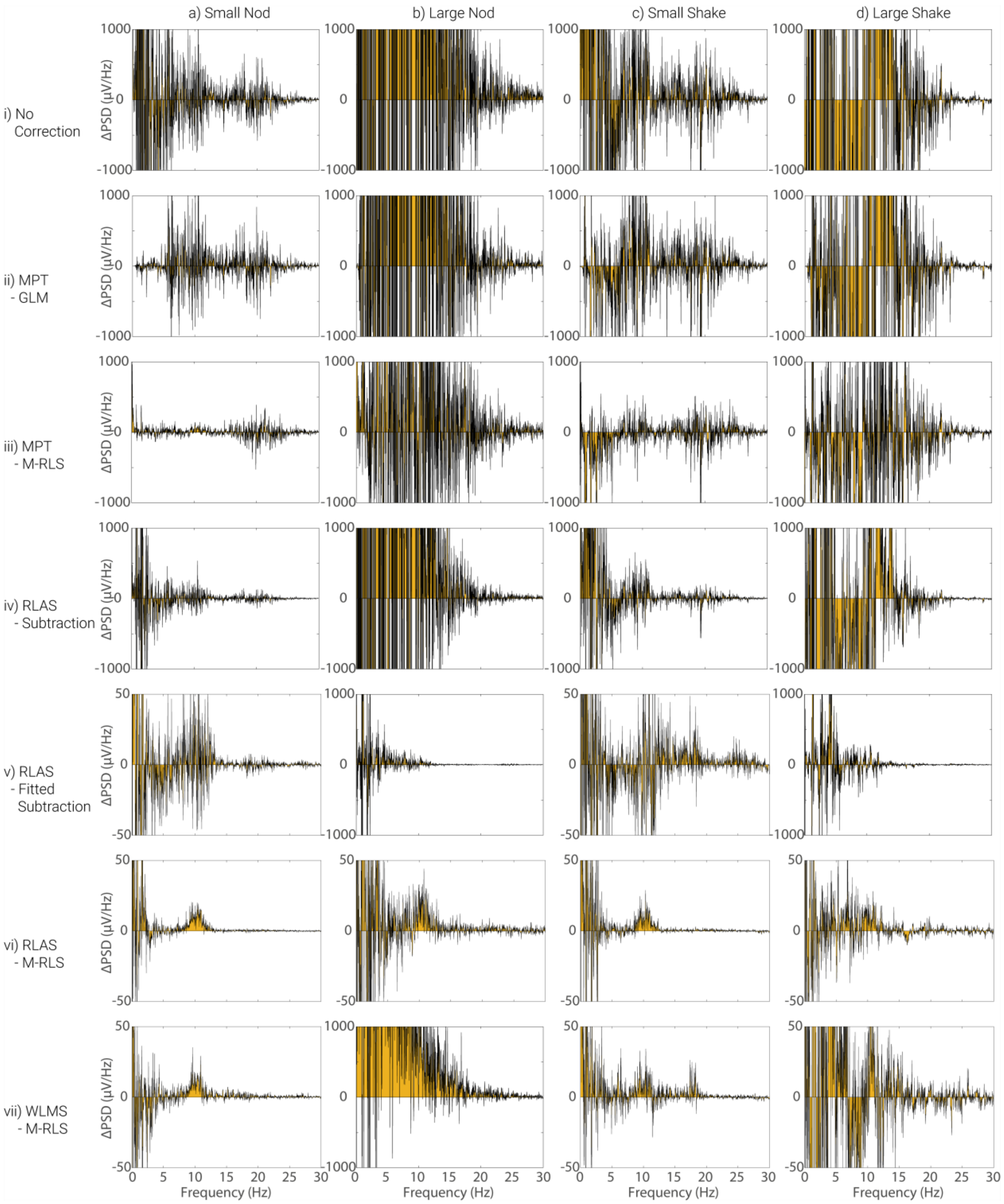

Figure S4: *The difference in the average power spectra from electrode O1 for eyes-open and eyes-closed conditions (generated from FFT's of open/closed response) where MAs have been added, row i, and subsequently corrected with different methods, rows ii-vii. MA data and motion recordings used for this figure are from dataset 2. Note the different scales in the spectra plotted across panels, and compared with Figure 4. Yellow shading denotes the area under the spectrum to aid visualisation.*

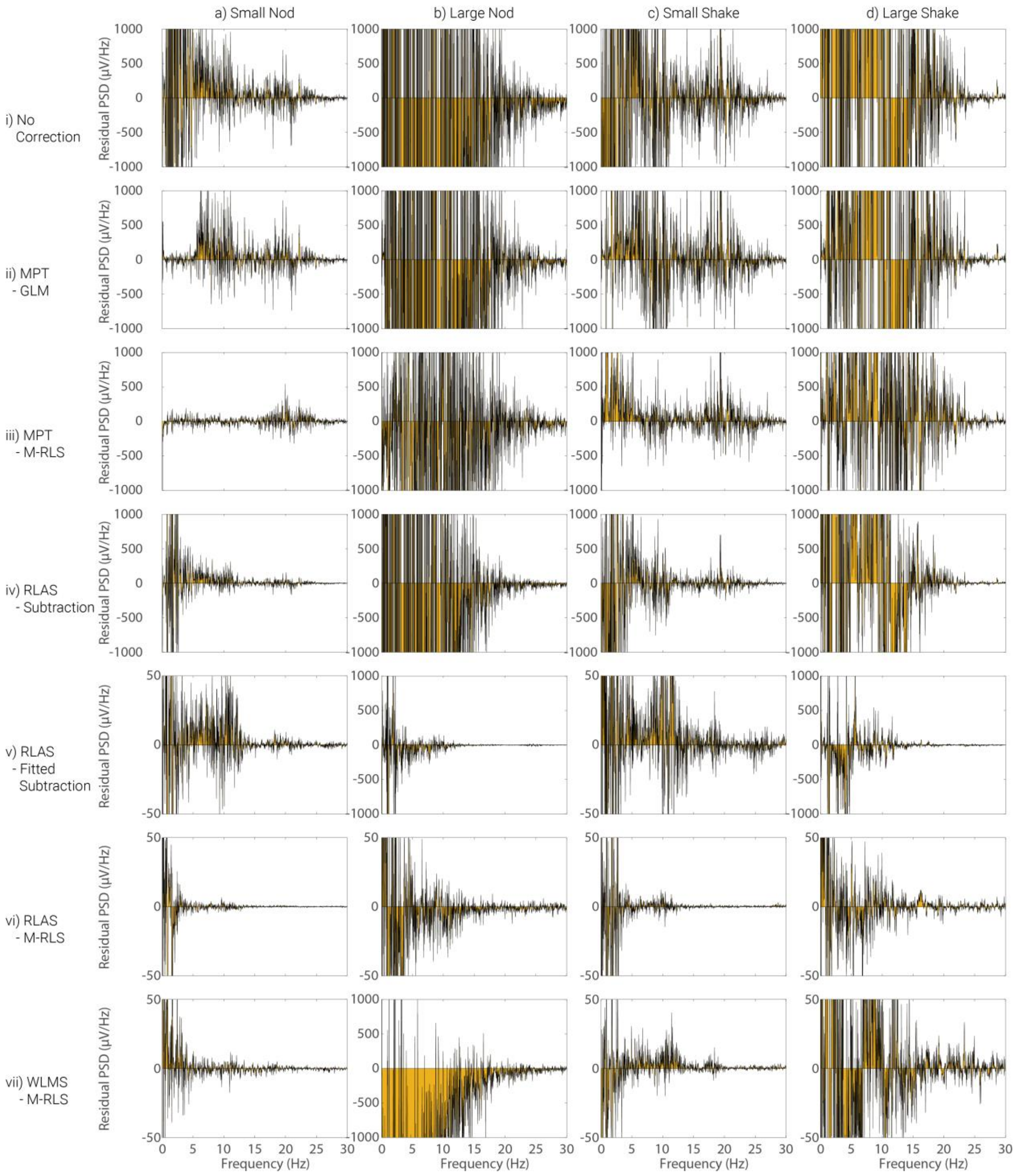

Figure S5: *The artefacts remaining after applying each correction method to eyes-open-eyes-closed (EOEC) data. The difference in the average power spectra from electrode O1 for eyes-open and eyes-closed conditions (generated from FFT's of open/closed response) where MAs have been added, row i, and subsequently corrected with different methods, rows ii-vii. In contrast to Fig. S4, here the gold-standard EOEC data (Fig. 4) has been subtracted, so only residual artefacts remain. MA data and motion recordings used for this figure are from dataset 2. Note the different scales in the spectra plotted across panels. Yellow shading denotes the area under the spectrum to aid visualisation.*

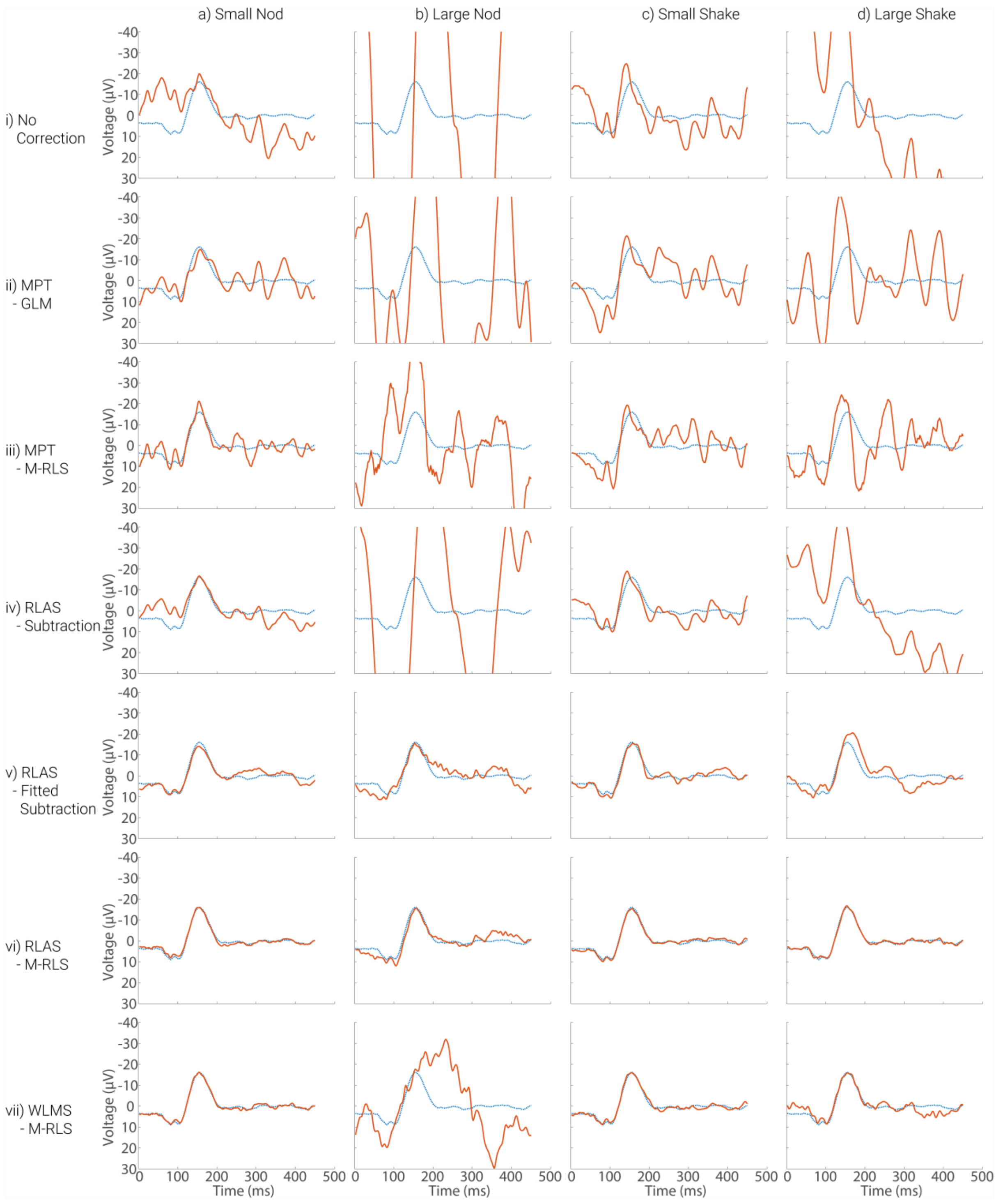

Figure S6: *The mean VEP measured from electrode O1, averaged over 240 trials. The mean gold standard VEP is shown by the blue line with the red lines showing responses with addition of MAs from dataset 2 (row i) and after MA correction using each of the methods (rows ii-vii).*

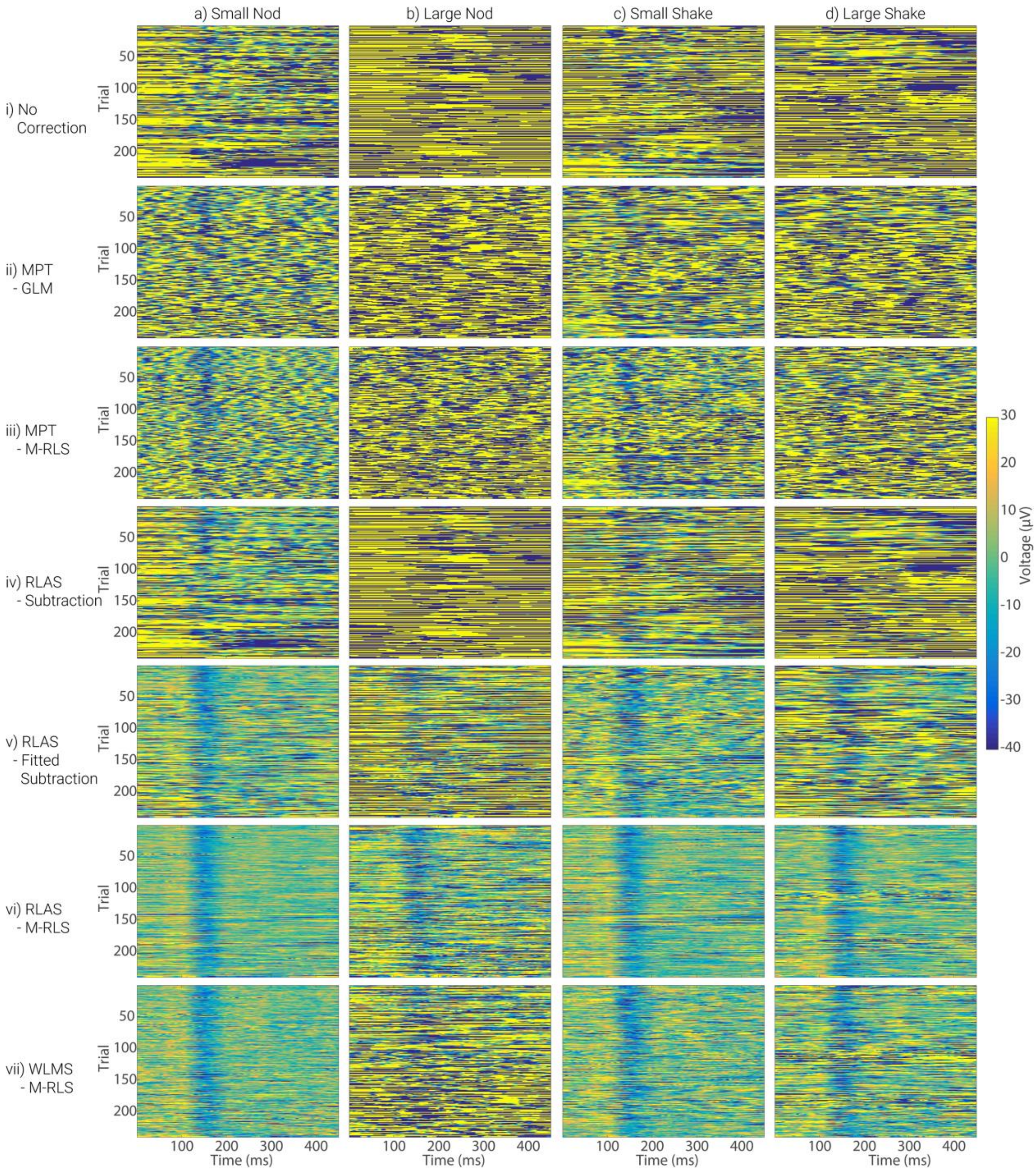

Figure S7: *The VEP signals measured from electrode O1 for each individual trial (y-axis) over the 450 ms period following stimulus onset (x-axis), with the MAs from dataset 2 added (row i). Rows ii-vii: show the responses that are revealed after each of the MA correction methods tested. Colour illustrates the voltage measured at each time point and in each trial.*

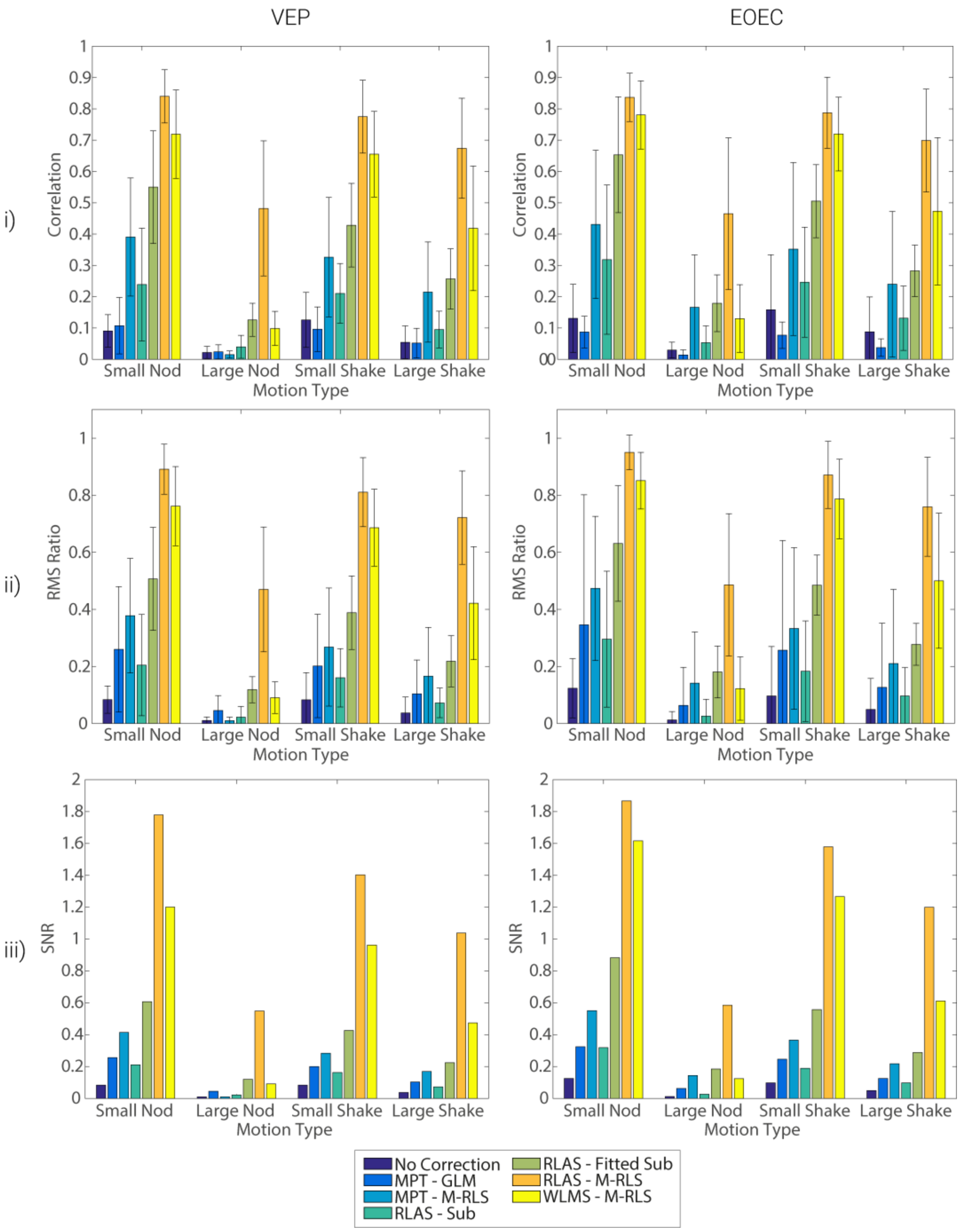

Figure S8: *Comparison over all electrodes of the relative performance of the different methods for correcting MA from EEG data, averaged over all electrodes. Comparisons are made for the evoked (VEP), left column, and oscillatory (eyes open/closed [EOEC]), right column, data. Metrics are derived for the neuronal response data combined with the MA data from dataset 2. Results with no MA correction are shown in dark blue and compared with each of the MA correction methods (see legend). Row i) shows the results from the correlation analysis, Row ii) shows the results from the RMS ratio analysis and Row iii) shows the outcome of the SNR analysis. Bars show the mean result over all electrodes on which MA data were recorded, whilst error bars denote the standard deviation of these metrics over electrodes. Standard deviations of SNR are shown separately, see Fig S6.*

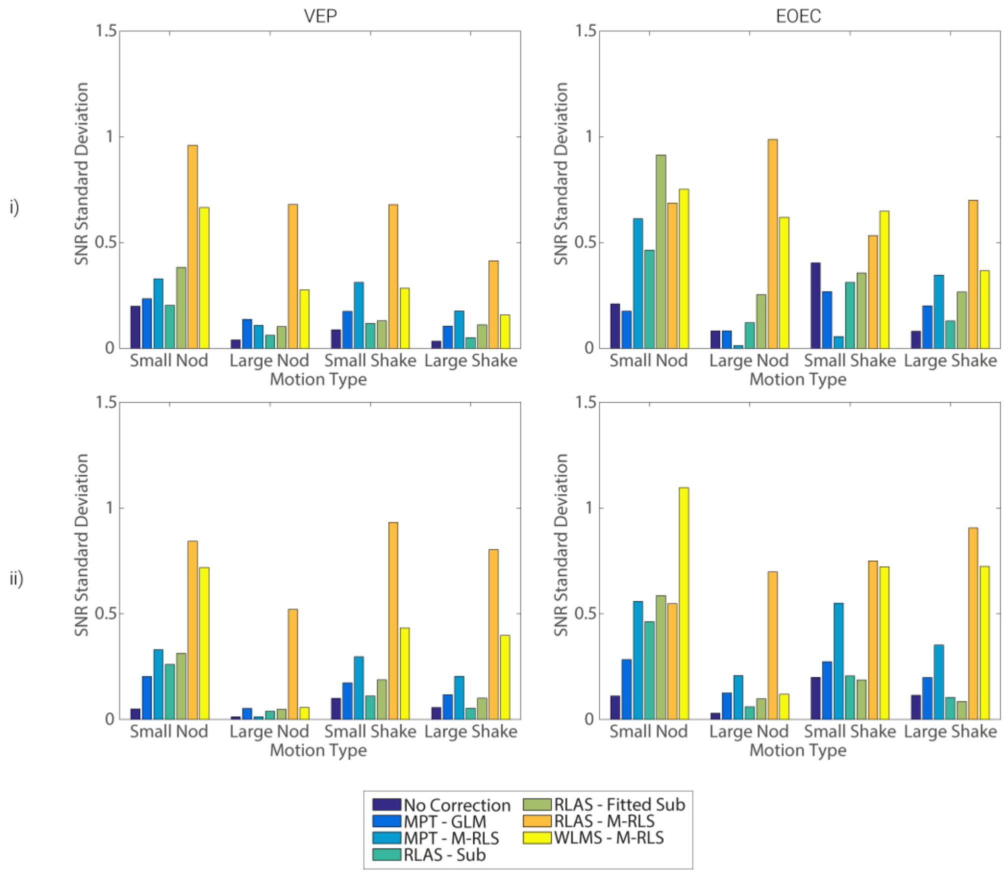

Figure S9: *Standard deviation of the SNR metrics across channels shown in Figures 9 and S5 for dataset 1 (row i) and dataset 2 (row ii). The large standard deviations show the large variability in SNR across the channels due to the neuronal signal to the visual stimulus being much larger in the occipital electrodes, as expected.*

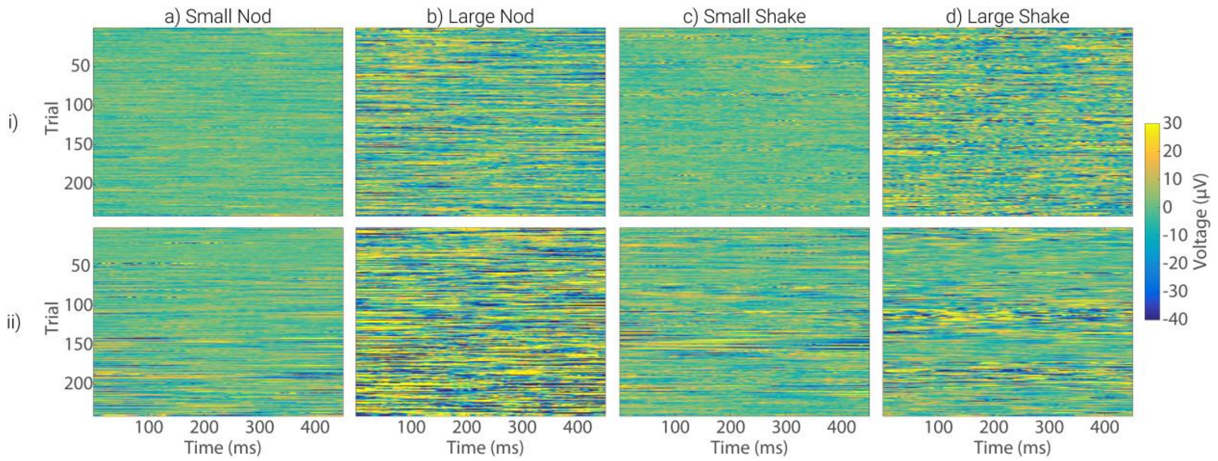

Figure S10: *Stack plots (as shown in Figures 7, 8 and S4) of the difference in the EEG data corrected for MA with the RLAS-M-RLS method (best performing method, row vi of Figs 8 and S4) and the original EEG data (i.e. the gold standard, Fig 7) for each of the movement types (columns) and dataset 1/2, (row i/ii respectively).*

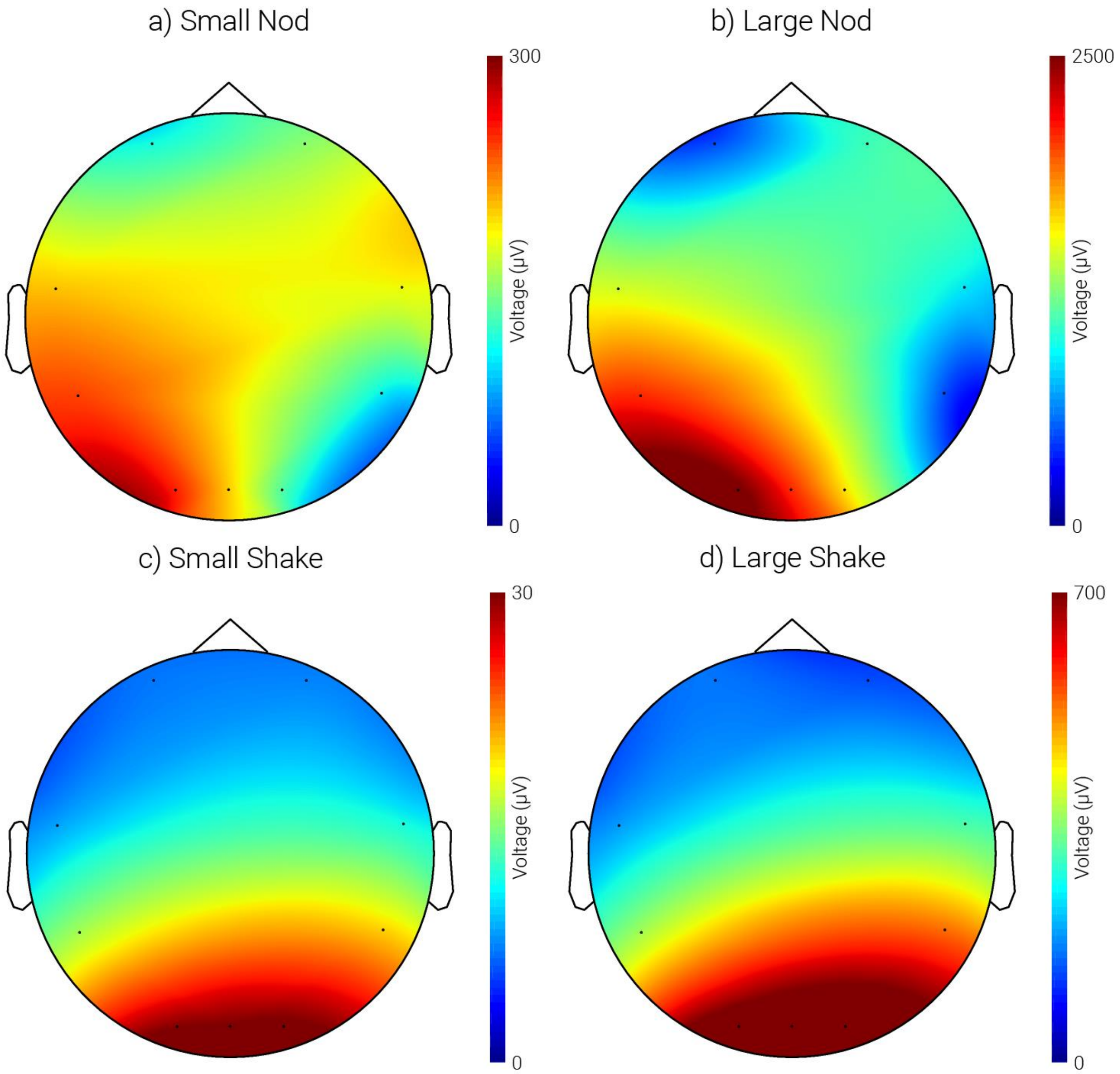

Figure S11: *The topography of the RMS of the MA recorded on the scalp electrodes for dataset 2.*

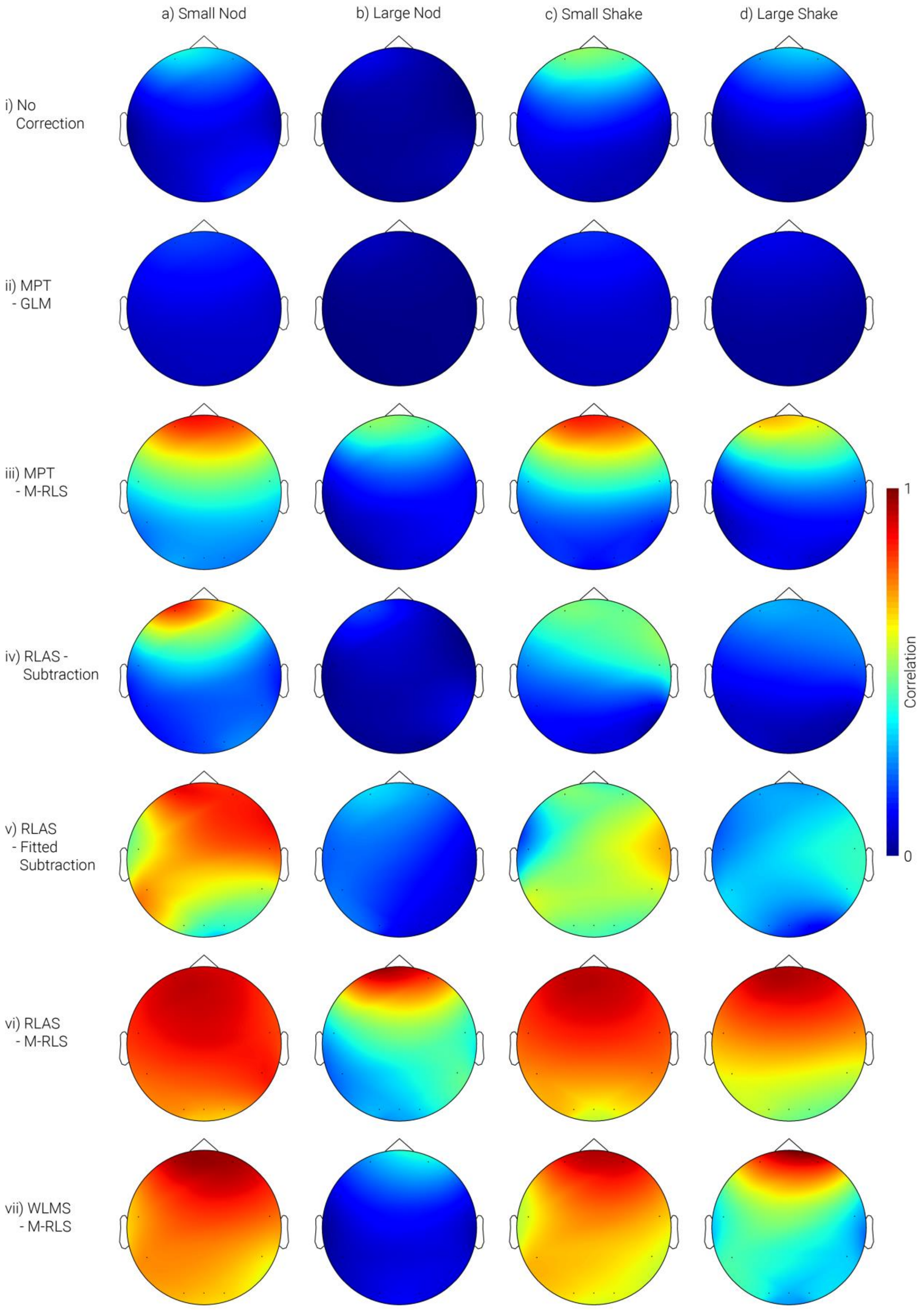

Figure S12: *The topography of the correlation of the corrected signal with the gold standard for each MA correction method applied to dataset 2.*

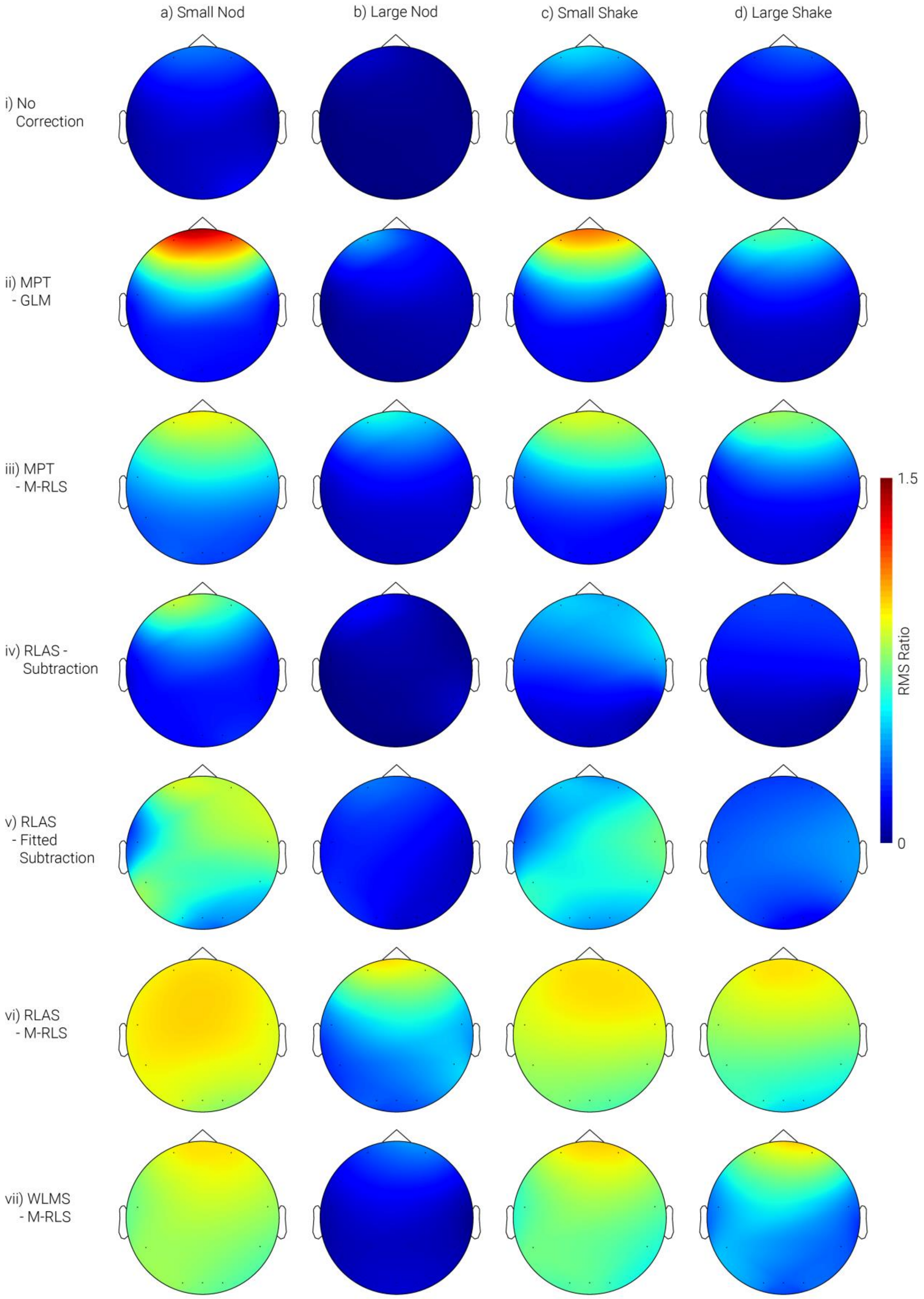

Figure S13: *The topography of the RMS ratio of the corrected signal for each MA correction method applied to dataset 2.*

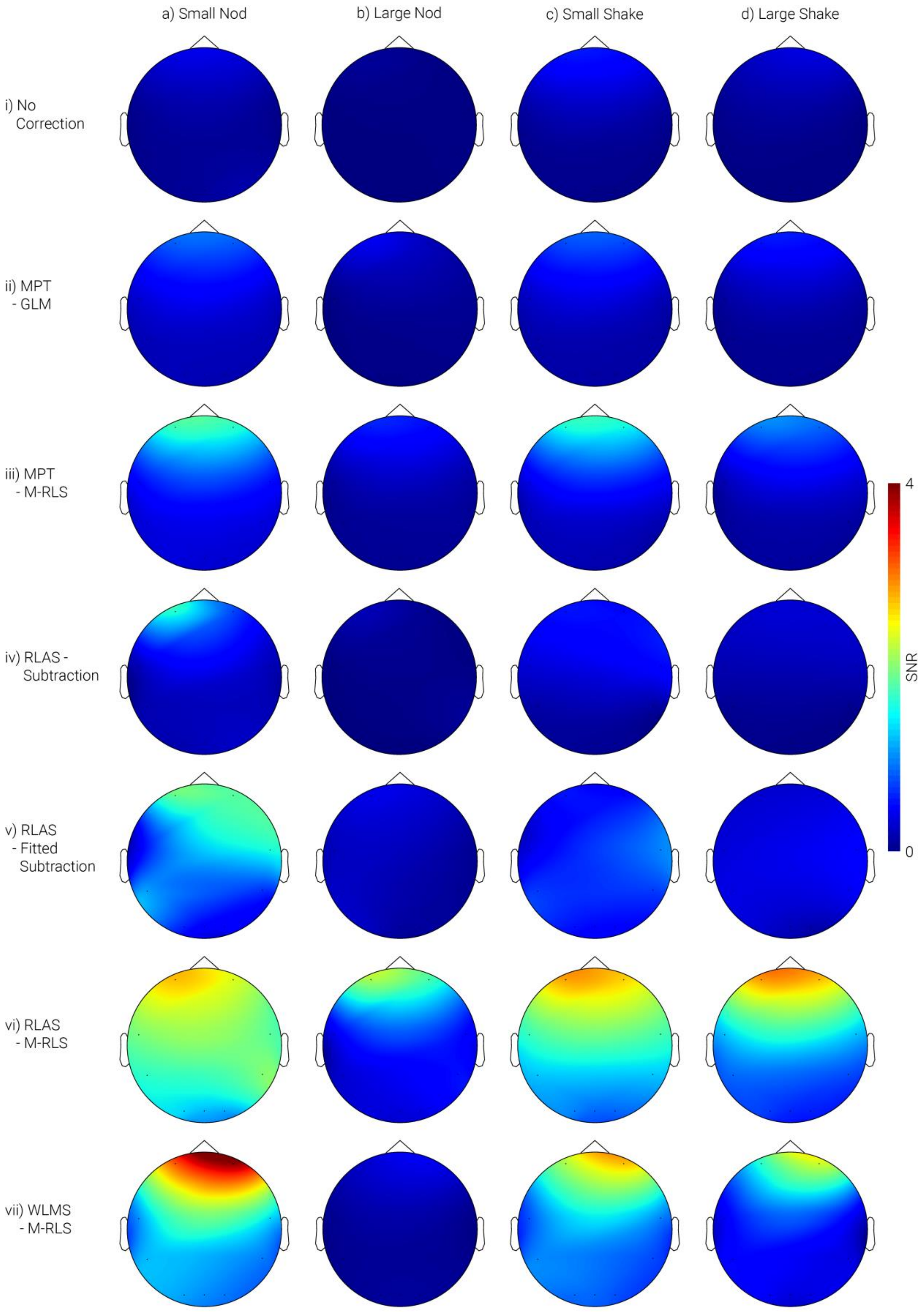

Figure S14: *The topography of the SNR of the corrected signal for each MA correction method applied to dataset 2.*
